# A CT-based deep learning model for the automated risk stratification of refractory *Mycoplasma pneumoniae* pneumonia in children

**DOI:** 10.1186/s12880-026-02590-y

**Published:** 2026-07-17

**Authors:** Zhoumeng Ying, Ge Hu, Jing Li, Wei Han, Fei Yang, Zheming Li, Zhenchen Zhu, Baofeng Zhang, Zhen Zhou, Weixiong Tan, Zhu Zhu, Mengyu Duan, Zhengyu Jin, Lan Song, Gang Yu

**Affiliations:** 1 Department of Radiology, State Key Laboratory of Complex Severe and Rare Diseases, Peking Union Medical College Hospital, Chinese Academy of Medical Sciences & Peking Union Medical College, Beijing, China; 2 4+4 Medical Doctor Program, Chinese Academy of Medical Sciences & Peking Union Medical College, Beijing, China; 3 Theranostics and Translational Research Center, National Infrastructures for Translational Medicine, Institute of Clinical Medicine, Peking Union Medical College Hospital, Chinese Academy of Medical Sciences & Peking Union Medical College, Beijing, China; 4 National Clinical Research Center for Child Health, Children’s Hospital, National Children’s Regional Medical Center, Zhejiang University School of Medicine, Hangzhou, 310052 China; 5 Sino-Finland Joint AI Laboratory for Child Health of Zhejiang Province, Hangzhou, China; 6 Department of Data and Information, Children’s Hospital, Zhejiang University School of Medicine, Hangzhou, China; 7 Department of Evidence-Based Medicine, Xuanwu Hospital, Capital Medical University, Beijing, China; 8 Department of CT, Rizhao Hospital of Traditional Chinese Medicine, Rizhao, China; 9 Department of Pediatrics, Rizhao Hospital of Traditional Chinese Medicine, Rizhao, China; 10 Deepwise AI Lab, Beijing Deepwise & League of PhD Technology Co.Ltd, Beijing, China; 11 Department of Radiology, Peking Union Medical College Hospital, Chinese Academy of Medical Sciences & Peking Union Medical College, No. 1 Shuaifuyuan, Dongcheng District, Beijing, 100730 People’s Republic of China; 12 National Clinical Research Center for Child Health, Children’s Hospital, Sino-Finland Joint AI Laboratory for Child Health of Zhejiang Province, National Children’s Regional Medical Center, Zhejiang University School of Medicine, No. 3333 Binsheng Road, Binjiang District, Hangzhou, 310000 People’s Republic Of China

**Keywords:** Pneumonia, Mycoplasma, Pediatrics, Deep learning, Computed tomography

## Abstract

**Background:**

The accurate identification of children with refractory *Mycoplasma pneumoniae* pneumonia (RMPP) remains challenging. This study aimed to develop a transformer-based model utilizing clinically indicated chest computed tomography (CT) to stratify pediatric RMPP risk at a critical decision point.

**Methods:**

Non-contrast chest CT data from a multicenter retrospective cohort of 1224 pediatric patients with *Mycoplasma pneumoniae* pneumonia who underwent clinically indicated CT were used to develop a transformer-based deep learning framework (trans-DLF). The primary cohort comprised training (*n* = 506), validation (*n* = 140), and internal testing (*n* = 139) cohorts, with two independent external cohorts (*n* = 331 and *n* = 108) used to evaluate generalizability. Model performance was assessed by the area under the receiver operating characteristic curve (AUC) and compared against a three-dimensional convolutional neural network (3D-CNN), a clinical model, and a multimodal nomogram. Interpretability was examined using gradient-weighted class activation mapping (Grad-CAM).

**Results:**

The median age was 6.83 years (interquartile range, 5.0–8.6 years), and 609 (49.8%) were male. The trans-DLF demonstrated strong performance across all cohorts: training (AUC 0.97; 95% confidence interval [CI], 0.96–0.98), validation (0.91; 0.86–0.96), internal testing (0.90; 0.85–0.95), and external testing (0.89; 0.84–0.94 and 0.89; 0.82–0.95). It significantly outperformed the clinical model (*p* < 0.001), while its AUCs were not significantly different from those of the multimodal nomogram. The model maintained good performance in outpatient settings (AUC 0.87) with good calibration and net clinical benefit. Grad-CAM suggested that predictions were influenced by clinically meaningful features, particularly consolidations.

**Conclusion:**

The trans-DLF provides a streamlined and efficient approach to RMPP risk assessment in children who have already undergone clinically indicated chest CT and may support timely, evidence-based decision-making without additional tests.

**Supplementary Information:**

The online version contains supplementary material available at https://doi.org/10.1186/s12880-026-02590-y.

## Background

*Mycoplasma pneumoniae* pneumonia (MPP) is a leading cause of pediatric community-acquired pneumonia, accounting for 10–40% of cases [1]. Although macrolides are the standard treatment, the rising prevalence of macrolide-resistant strains, particularly in East Asia, has created a major clinical challenge [2, 5]. Consequently, a substantial proportion of children (12.8–52.2%) develop refractory MPP (RMPP), characterized by persistent fever and radiological progression despite treatment [6, 10]. RMPP frequently necessitates the use of second-line antibiotics or immunomodulators and is associated with an increased risk of severe, long-term sequelae, including pulmonary necrosis and obliterative bronchiolitis [7, 11–15]. Thus, the accurate identification of pediatric RMPP is crucial for guiding timely clinical intervention.

However, the accurate and timely identification of RMPP remains challenging [11, 12]. While chest radiography is the standard first-line imaging modality for pediatric MPP, computed tomography (CT) is frequently necessitated for patients who exhibit clinical non-improvement or suspected severe parenchymal involvement [2, 16, 20]. In this specific cohort, CT offers superior anatomic resolution, and the adoption of low-dose protocols has effectively mitigated radiation concerns [21, 22]. Furthermore, specific CT features have emerged as independent predictors of RMPP, establishing the modality’s dual role as both a diagnostic and prognostic tool for patients at risk of RMPP [10, 23, 26].

After a CT scan is performed, clinicians must rapidly determine the probability of RMPP to support timely clinical reassessment, intensified monitoring, and consideration of treatment escalation in conjunction with the overall clinical context. In this acute clinical setting, although various RMPP prediction models have been developed, most current models are multimodal and rely on time-intensive laboratory results, manual data entry, subjective radiological interpretation, or complex image preprocessing [26, 30]. These limitations impede clinical deployment and fail to meet the requirements for timely clinical decision support in acute pediatric care settings. As a result, there remains a clinical need for a streamlined, time-efficient risk stratification model for patients awaiting immediate management.

Unimodal CT-based deep learning (DL) approaches can offer a viable solution to this problem. However, because different neural network architectures capture spatial information in different ways, model architecture may influence performance and clinical utility. While convolutional neural networks (CNNs) have historically dominated medical imaging and are effective for learning local spatial patterns [31], transformer-based architectures leverage self-attention to capture broader contextual information [32]. By processing the entire lung volume as a unified input, a transformer-based framework can autonomously extract complex, high-dimensional features directly from raw images, thereby enhancing objectivity, reproducibility, and translational potential.

This study presents a transformer-based deep learning framework (trans-DLF) for automated and efficient risk stratification of RMPP using baseline non-contrast chest CT. For patients undergoing CT because of clinical concern, the framework provides rapid risk stratification at the point of imaging and may support timely clinical reassessment and intervention.

## Methods

This study was conducted in accordance with the Declaration of Helsinki and approved by the institutional review boards of all participating hospitals (2024-IRB-0189-P-01, 2024-IRB-039, and 1-23PJ078). The requirement for individual informed consent was waived due to the retrospective design. All DICOM images and clinical data were de-identified before analysis. Access to the study data was restricted to authorized members of the research team, and no directly identifiable patient information was used for model development or statistical analysis.

### Study design and participants

**Fig. 1:**
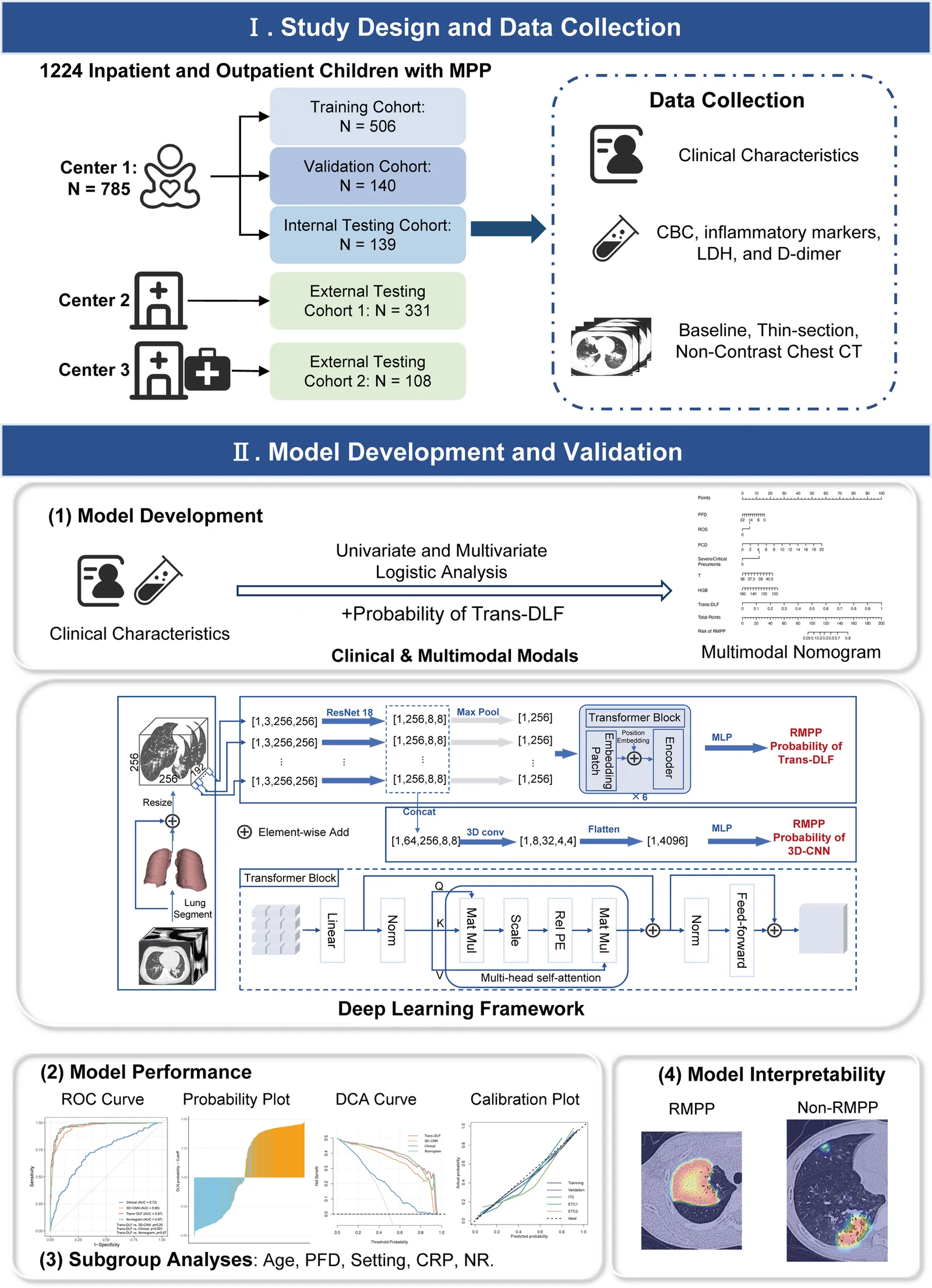
The flowchart of the study. Abbreviations: CT, computed tomography; CBC, complete blood count; LDH, lactate dehydrogenase; RMPP, refractory mycoplasma pneumoniae pneumonia; MLP, multilayer perceptron; RelPE, relative positional encoding; Trans-DLF, Transformer-based deep learning framework; 3D-CNN, 3D convolutional neural network; CRP, C-reactive protein; NR, neutrophil ratio; PFD, pre-visit fever duration; ROC, receiver-operator characteristic; DCA, decision curve analysis

**Fig. 2:**
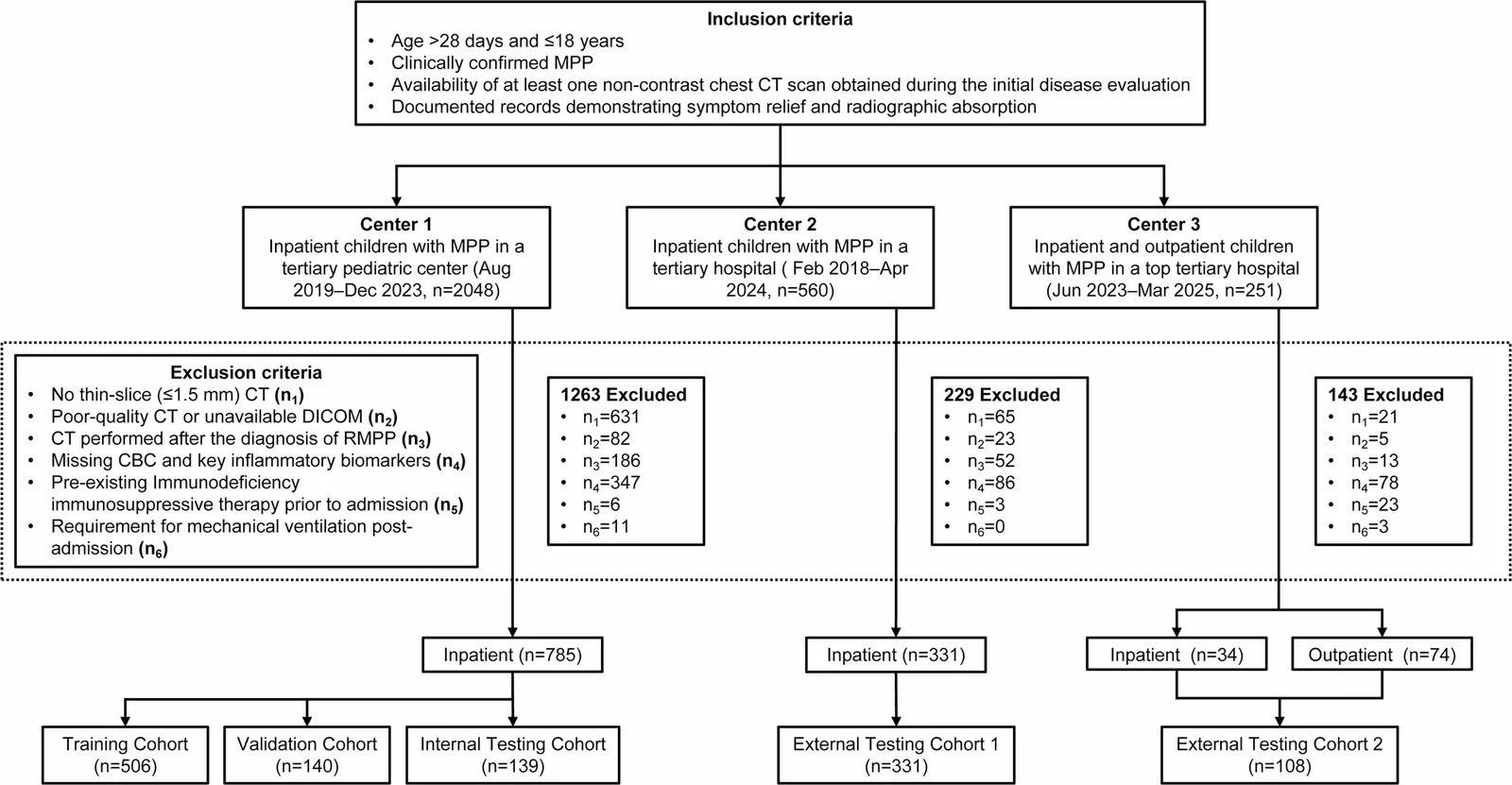
Overview of patient enrollment, exclusion criteria, and dataset construction. Abbreviations: MPP, *mycoplasma pneumoniae* pneumonia; RMPP, refractory *mycoplasma pneumoniae* pneumonia; CT, computed tomography; CBC, complete blood count

This retrospective, multi-center study consecutively enrolled pediatric patients with MPP from three hospitals, each representing different levels of healthcare. The development dataset, which was established using data from Center 1, a national pediatric referral center, consisted of 785 inpatients enrolled between August 2019 and December 2023. These patients were randomly divided into a training cohort (*n* = 506), a validation cohort (*n* = 140), and an internal testing cohort (*n* = 139) at an approximate allocation ratio of 3:1:1. Each patient contributed only one baseline CT examination, and no patient overlap occurred among the three cohorts. To assess generalizability, external validation was performed using two independent cohorts: External cohort 1 consisted of 331 patients enrolled between February 2018 and April 2024 at Center 2, a regional tertiary hospital, and external cohort 2 consisted of 108 inpatients and outpatients enrolled between June 2023 and March 2025 at Center 3, a large comprehensive general hospital. The overall flowchart and study flow diagram are depicted in Figs. 1 and 2.

The inclusion criteria were: (1) age > 28 days and ≤ 18 years; (2) clinically confirmed MPP; (3) availability of at least one non-contrast chest CT scan obtained during the initial disease evaluation; and (4) documented records of symptom resolution and radiographic absorption. The exclusion criteria were: (1) no thin-slice (≤ 1.5 mm) CT; (2) poor-quality CT or unavailable Digital Imaging and Communications in Medicine (DICOM) data; (3) chest CT performed after the RMPP diagnosis; (4) missing complete blood count (CBC) and key inflammatory biomarkers; (5) pre-existing immunodeficiency or immunosuppressive therapy prior to admission; and (6) requirement for mechanical ventilation post-admission. The diagnostic criteria for MPP are detailed in Supplementary Material 1.1.

### Clinical and radiologic data collection

Thin-section (≤ 1.5 mm), non-contrast chest CT scans and corresponding CT reports were retrieved from each hospital’s institutional Picture Archiving and Communication System. For all patients, the first available baseline CT scan acquired prior to RMPP diagnosis was retrieved. CT examinations were performed at the discretion of treating pediatricians when guideline-based indications were met. These indications included concern for severe pulmonary involvement (such as extensive consolidation, necrosis, or pleural effusion), a refractory clinical course after 48–72 h of targeted therapy, the need to exclude alternative diagnoses, discordance between clinical presentation and chest radiography, or evaluation of recurrent pneumonia [2, 16, 20]. Detailed CT acquisition protocols across the centers are summarized in Supplementary Table S1.

Demographic, clinical, and laboratory data, including CBC and inflammatory markers, including C-reactive protein (CRP), lactate dehydrogenase (LDH), and D-dimer levels, were collected from the electronic hospital information system. Clinical and laboratory data were collected at admission for inpatients and at the initial visit for outpatients. All analyzed variables and their proportions of missing data are listed in Supplementary Table S2.

RMPP was defined as the presence of persistent fever, worsening clinical symptoms, radiologic progression, or extrapulmonary complications after at least seven days of macrolide therapy [11]. Radiographic progression in RMPP was defined as interval worsening on follow-up imaging after adequate macrolide therapy. Disease severity was categorized as mild or severe/critical according to the 2023 Chinese pediatric MPP guidelines [2], as detailed in Supplementary Material 1.2. Classification of both RMPP status and disease severity was performed by an experienced pediatric pulmonologist.

### Construction of the DL-based models

Trans-DLF was developed as an automated warning system for rapid RMPP risk stratification by extracting comprehensive global features from the entire lung volume. Following automated lung segmentation and grouping CT slices into three-slice blocks, local features were then extracted from each block using the ResNet-18 encoder. These slice-level representations were then input into a six-layer transformer encoder equipped with multi-head self-attention and relative positional encoding, allowing the model to capture long-range and global inter-slice dependencies across the whole lung. Finally, aggregated features from the transformer were subsequently processed by a multilayer perceptron to generate patient-level probabilities of RMPP. For comparison, a three-dimensional convolutional neural network (3D-CNN) was constructed as a baseline to compare transformer-based modeling with a conventional volumetric convolutional approach. Further details about the model construction are provided in Fig. 1 and Supplementary Material 1.3.

### Construction of the clinical model and multimodal nomogram

To ensure data quality, variables with more than 40% missingness across all datasets were excluded. The remaining missing values were imputed independently for each cohort using predictive mean matching to prevent data leakage. For the primary analysis, one completed dataset was randomly selected from 20 imputed datasets for clinical model development and evaluation. Because this approach did not account for between-imputation variability, we additionally performed a sensitivity analysis using all 20 imputed datasets, as detailed in Supplementary Material 1.4. The cohort-specific missingness rates for all candidate variables are reported in Supplementary Table S2. Potential clinical predictors with a *p* value of less than 0.05 in the univariate logistic regression analyses were entered into a multivariable logistic regression model. Predictors that remained statistically significant in the multivariable analysis were retained in the final clinical model. These predictors were subsequently integrated with the trans-DLF probability to develop a multimodal nomogram.

### Model performance and clinical utility evaluation

Model performance was primarily assessed by calculation of the area under the receiver operating characteristic curve (AUC). The optimal threshold was determined by maximizing Youden’s index in the validation cohort. Within each cohort, the AUC of Trans-DLF was compared with those of the clinical model, the 3D-CNN model, and the nomogram, and the resulting *p* values were adjusted for the three pairwise comparisons using the Bonferroni method. Secondary metrics, including accuracy, sensitivity, specificity, positive predictive value (PPV), and negative predictive value (NPV), were determined using a stratified bootstrapping procedure with 2000 replicates and reported with their 95% confidence intervals (CIs). Significant differences between model AUCs were assessed using DeLong’s test [33]. Model calibration, representing the concordance between predicted probabilities and observed outcomes, was evaluated with calibration curve analysis using the “rms” package in R, with ideal calibration considered close alignment with the 45° line. Clinical utility was estimated using decision curve analysis (DCA) to quantify the net benefit at various threshold probabilities.

To assess robustness, subgroup analyses were conducted by stratifying patients by age, disease severity, pre-visit fever duration (PFD), treatment setting, and initial levels of CRP and neutrophil ratio (NR). Stratification cutoffs for laboratory biomarkers were identified via receiver operating characteristic curve analysis and Youden’s index in the validation cohort, with age cutoffs based on the cohort age distribution (Figure S1) and known MPP epidemiology [34]. To enhance model interpretability, gradient-weighted class activation mapping (Grad-CAM) was used to visualize key regions of the CT scans most influential for risk stratification [35].

In addition to the primary threshold selected by maximizing the Youden index, we explored two clinically oriented operating points for the trans-DLF. In the validation cohort, the sensitivity-oriented threshold was the highest cutoff achieving sensitivity of at least 0.90, whereas the specificity-oriented threshold was the lowest cutoff achieving specificity of at least 0.90.

### Statistical analysis

All statistical analyses were performed using R software (version 4.3.1). Continuous variables were compared using the Student’s *t*-test or the Mann–Whitney U-test, and categorical variables were compared using the chi-square or Fisher’s test, as appropriate. A two-tailed *p*-value < 0.05 was considered statistically significant. Reporting of this study was guided by the Completeness, Learnability, Applicability, Interpretability, Reproducibility, and Evaluation framework [36]. The completed checklist is provided in Supplementary Table S3.

## Results

### Characteristics of the study population

A total of 1224 pediatric patients, 538 with RMPP and 686 with non-RMPP, were enrolled in this multicenter study, corresponding to an overall RMPP frequency of 43.95%. Their baseline demographic and clinical characteristics are summarized in Table 1 and Supplementary Tables S4 and S5. The overall cohort had a median age of 6.83 years (interquartile range [IQR], 5.0–8.6) and a balanced sex distribution (609 males, 49.8%; 615 females, 50.2%). The baseline CT was performed a median of 7 days (IQR, 5–9) after symptom onset and 4 days (IQR, 3–6) after the initiation of standard macrolide therapy.

**Table 1:** Demographic and clinical characteristics of patients with refractory and non-refractory mycoplasma pneumonia pneumonia in center 1

Variables	Training cohort (*n* = 506)			Validation cohort (*n* = 140)			Internal testing cohort (*n* = 139)		
	Non-RMPP (*n* = 254)	RMPP (*n* = 252)	*p*-value	Non-RMPP (*n* = 71)	RMPP (*n* = 69)	*p*-value	Non-RMPP (*n* = 70)	RMPP (*n* = 69)	*p*-value
Sex (%)			0.66			0.40			0.93
Male	133 (52.36)	126 (50.00)		32 (45.07)	37 (53.62)		36 (51.43)	34 (49.28)	
Female	121 (47.64)	126 (50.00)		39 (54.93)	32 (46.38)		34 (48.57)	35 (50.72)	
Age, years (median [IQR])	6.79 [4.90, 8.00]	6.25 [4.23, 8.10]	0.07	5.83 [3.92, 7.62]	6.33 [4.50, 8.25]	0.31	5.42 [3.85, 7.02]	6.42 [4.75, 8.08]	0.03*
Comorbidity (%)	24 (9.45)	32 (12.70)	0.31	9 (12.68)	10 (14.49)	0.95	11 (15.71)	4 (5.80)	0.11
PFD, days (median [IQR])	6.00 [4.00, 7.00]	7.00 [5.00, 9.00]	< 0.001*	5.00 [4.00, 7.00]	7.00 [5.00, 9.00]	< 0.001*	5.00 [4.00, 7.00]	7.00 [5.00, 9.00]	< 0.001*
PCD, days (median [IQR])	5.00 [4.00, 7.00]	7.00 [5.00, 9.00]	< 0.001*	5.00 [3.00, 6.00]	7.00 [5.00, 9.00]	< 0.001*	5.00 [4.00, 7.00]	6.00 [5.00, 8.00]	0.008*
Severe/Critical Pneumonia (%)	196 (77.17)	225 (89.29)	< 0.001*	50 (70.42)	60 (86.96)	0.03*	53 (75.71)	63 (91.30)	0.03*
ROS (%)	15 (5.91)	49 (19.44)	< 0.001*	4 (5.63)	14 (20.29)	0.02*	3 (4.29)	19 (27.54)	< 0.001*
T, ℃ (median [IQR])	37.20 [36.70, 37.90]	37.50 [36.90, 38.40]	0.003*	37.40 [36.80, 38.20]	37.60 [36.70, 38.40]	0.63	37.20 [36.80, 37.65]	37.65 [37.10, 38.38]	0.004*
P, beats/min (mean (SD))	120.00 [110.00, 132.00]	124.00 [116.00, 136.00]	0.002*	120.00 [108.00, 132.00]	124.00 [117.50, 132.00]	0.21	120.00 [111.50, 130.00]	124.00 [118.00, 136.00]	0.07
R, breaths/min (median [IQR])	28.00 [24.00, 32.00]	30.00 [26.00, 34.00]	< 0.001*	30.00 [26.00, 32.00]	30.00 [26.00, 36.00]	0.64	30.00 [26.00, 32.00]	30.00 [26.00, 32.00]	0.94

Note: Unless otherwise indicated, data are presented as the number of patients, with percentages in parentheses

Abbreviations: RMPP, refractory *mycoplasma pneumoniae* pneumonia; IQR, Interquartile range; PFD, pre-visit fever duration; PCD, pre-visit cough duration; ROS, requirement for oxygen support; T, temperature; P, pulse rate; R, respiratory rate. *Represents *p* < 0.05

Multivariable logistic regression in the training cohort identified six independent indicators of RMPP (all *p* < 0.05, Table 2). These variables and the probability from the trans-DLF were then incorporated into a multimodal nomogram (Figure S2). The multiple-imputation sensitivity analysis largely confirmed the primary findings, with five of the six predictors remaining statistically significant. Hemoglobin, which showed only borderline significance in the primary analysis, was no longer significant after pooling estimates across the 20 imputed datasets (Table S6 and Fig. S3).

**Table 2:** Univariate and multivariable logistic regression analysis of clinical features associated with RMPP in the training cohort

Variables	Univariate analysis		Multivariate analysis	
	OR (95% CI)	*p*-value	*OR (95% CI)*	*p*-value
Sex	0.91 (0.64–1.29)	0.60		
Age	0.94 (0.87–1.00)	0.06		
Comorbidity	1.39 (0.80–2.46)	0.25		
PFD	1.18 (1.10–1.27)	< 0.001*	1.12 (1.03–1.23)	0.01*
PCD	1.13 (1.07–1.20)	< 0.001*	1.09 (1.02–1.18)	0.02*
Severe/Critical Pneumonia	2.47 (1.52–4.10)	< 0.001*	1.89 (1.11–3.29)	0.02*
ROS	3.85 (2.14–7.29)	< 0.001*	3.23 (1.69–6.47)	0.001*
T	1.34 (1.10–1.63)	0.003*	1.43 (1.12–1.82)	0.004*
P	1.02 (1.01–1.03)	0.001*	1.00 (0.99–1.01)	0.96
R	1.05 (1.02–1.09)	0.001*	1.01 (0.97–1.05)	0.59
CRP	1.01 (1.00–1.02)	0.02*	1.01 (1.00–1.02)	0.07
WBC	1.08 (1.02–1.14)	0.007*	1.03 (0.97–1.09)	0.36
NR	1.01 (1.00–1.02)	0.17		
MR	0.99 (0.93–1.06)	0.87		
LR	0.99 (0.97–1.00)	0.13		
BR	1.70 (0.64–4.64)	0.29		
ER	1.02 (0.94–1.10)	0.69		
HGB	0.97 (0.96–0.99)	0.01*	0.98 (0.96–1.00)	0.048*
PLT	1.00 (1.00–1.00)	0.30		
Atelectasis	0.87 (0.59–1.27)	0.47		
Pleural effusion	1.63 (1.10–2.43)	0.02*	1.29 (0.83–2.00)	0.25

Note: Only variables that demonstrated statistical significance in the univariate analysis were subsequently included in the multivariable analysis

Abbreviations: OR, Odds ratio; CI, confidence interval; RMPP, refractory mycoplasma pneumoniae pneumonia; PFD, pre-visit fever duration; PCD, pre-visit cough duration; ROS, requirement for oxygen support; T, temperature; P, pulse rate; R, respiratory rate; CRP, C-reactive protein; WBC, white blood cell count; NR, neutrophil ratio; MR, monocyte ratio; LR, lymphocyte ratio; BR, basophil ratio; ER, eosinophil ratio; HGB, hemoglobin; PLT, platelet count. *Represents *p* < 0.05

### Model performance

**Fig. 3:**
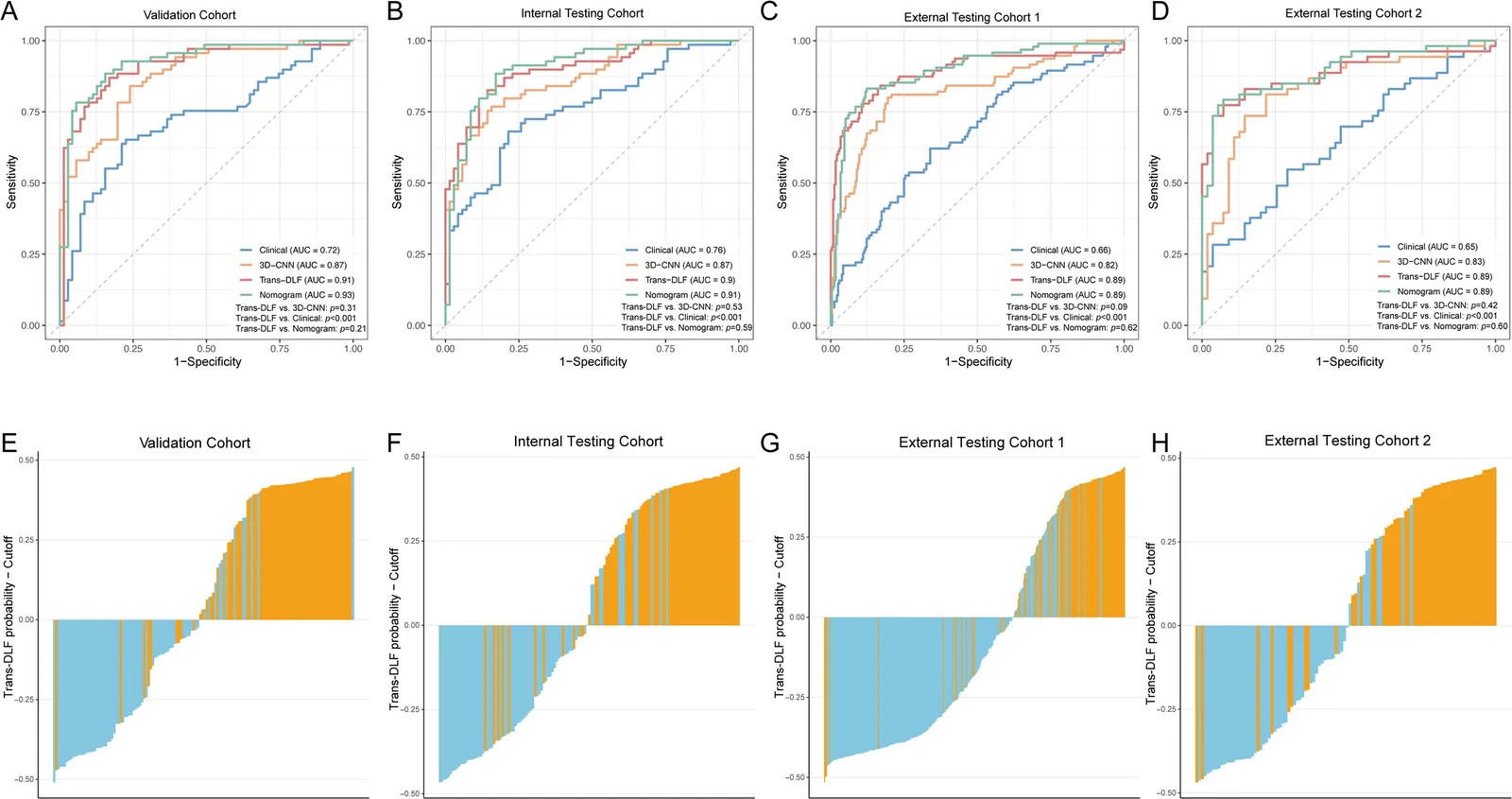
Risk stratification performance for refractory *Mycoplasma pneumoniae* pneumonia (RMPP). (**A**–**D**) Receiver operating characteristic (ROC) curves comparing the transformer-based deep learning framework (trans-DLF), the 3D convolutional neural network (3D-CNN), the clinical model, and the multimodal nomogram in the validation cohort, internal testing cohort, and two external testing cohorts. (**E**–**H**) Waterfall plots illustrating the predicted probabilities of RMPP generated by the trans-DLF in the corresponding cohorts. An optimal cutoff of 0.5174 (the horizontal baseline) was used to classify cases. Blue bars below the baseline represent correctly classified non-RMPP cases. Orange bars above the baseline indicate correctly classified RMPP cases. Bars that cross the baseline represent misclassified cases. Abbreviations: Trans-DLF, Transformer-based deep learning framework; 3D-CNN, 3D convolutional neural network; AUC, area under the curve

The proposed trans-DLF model demonstrated strong discrimination for RMPP in all cohorts (Figs. 3 and S4, Table 3, and Supplementary Tables S7 and S8). In the training cohort, the trans-DLF model achieved an AUC of 0.97 (95% CI, 0.96–0.98), with an accuracy of 0.92, sensitivity of 0.96, and specificity of 0.87. This high performance was maintained in the validation cohort, with an AUC of 0.91 (95% CI, 0.86–0.96), and in the internal testing cohort, with an AUC of 0.90 (95% CI, 0.85–0.95). Importantly, the trans-DLF model exhibited good generalizability, achieving AUCs of 0.89 (95% CI, 0.84–0.94) and 0.89 (95% CI, 0.82–0.95) in external testing cohorts 1 and 2, respectively.

**Table 3:** Predictive performance of the deep learning and clinical models for refractory mycoplasma pneumoniae pneumonia

Models	AUC (95% CI)	ACC (95% CI)	SEN (95% CI)	SPE (95% CI)	PPV (95% CI)	NPV (95% CI)	F1 Score (95% CI)
Trans-DLF							
Training	0.97 (0.96–0.98)	0.92 (0.89–0.94)	0.96 (0.94–0.98)	0.87 (0.83–0.91)	0.88 (0.84–0.92)	0.96 (0.93–0.98)	0.92 (0.89–0.94)
Validation	0.91 (0.86–0.96)	0.85 (0.79–0.91)	0.87 (0.79–0.94)	0.83 (0.73–0.91)	0.83 (0.74–0.92)	0.87 (0.79–0.94)	0.85 (0.79–0.91)
ITC	0.90 (0.85–0.95)	0.83 (0.76–0.89)	0.84 (0.75–0.92)	0.81 (0.72–0.90)	0.82 (0.72–0.90)	0.84 (0.75–0.92)	0.83 (0.75–0.89)
ETC1	0.89 (0.84–0.94)	0.82 (0.78–0.86)	0.84 (0.76–0.91)	0.81 (0.76–0.86)	0.65 (0.56–0.73)	0.93 (0.89–0.96)	0.73 (0.66–0.79)
ETC2	0.89 (0.82–0.95)	0.83 (0.76–0.90)	0.83 (0.72–0.92)	0.84 (0.73–0.93)	0.83 (0.73–0.93)	0.84 (0.74–0.93)	0.83 (0.74–0.90)
3D-CNN							
Training	0.95 (0.94–0.97)	0.79 (0.75–0.83)	0.99 (0.98–1.00)	0.59 (0.53–0.65)	0.71 (0.66–0.75)	0.99 (0.97–1.00)	0.83 (0.79–0.86)
Validation	0.87 (0.82–0.93)	0.80 (0.73–0.86)	0.84 (0.75–0.92)	0.76 (0.66–0.86)	0.77 (0.68–0.87)	0.83 (0.73–0.92)	0.81 (0.73–0.87)
ITC	0.87 (0.81–0.93)	0.69 (0.61–0.77)	0.88 (0.80–0.96)	0.50 (0.39–0.63)	0.64 (0.54–0.73)	0.81 (0.69–0.93)	0.74 (0.66–0.81)
ETC1	0.82 (0.77–0.88)	0.80 (0.76–0.85)	0.78 (0.69–0.86)	0.81 (0.77–0.86)	0.63 (0.54–0.71)	0.90 (0.86–0.94)	0.69 (0.62–0.76)
ETC2	0.83 (0.75–0.91)	0.77 (0.69–0.84)	0.75 (0.64–0.86)	0.78 (0.67–0.89)	0.77 (0.65–0.88)	0.77 (0.65–0.88)	0.76 (0.67–0.84)
Clinical							
Training	0.72 (0.67–0.76)	0.67 (0.63–0.71)	0.58 (0.52–0.64)	0.75 (0.70–0.80)	0.70 (0.64–0.76)	0.65 (0.58–0.70)	0.64 (0.58–0.69)
Validation	0.72 (0.64–0.81)	0.71 (0.64–0.79)	0.65 (0.53–0.76)	0.78 (0.68–0.87)	0.74 (0.62–0.85)	0.70 (0.59–0.80)	0.69 (0.59–0.78)
ITC	0.76 (0.68–0.84)	0.73 (0.66–0.81)	0.68 (0.56–0.79)	0.79 (0.68–0.88)	0.76 (0.64–0.86)	0.71 (0.61–0.81)	0.72 (0.61–0.80)
ETC1	0.66 (0.60–0.73)	0.71 (0.66–0.76)	0.27 (0.19–0.36)	0.88 (0.84–0.92)	0.48 (0.35–0.61)	0.75 (0.70–0.80)	0.35 (0.25–0.44)
ETC2	0.65 (0.55–0.76)	0.58 (0.49–0.67)	0.30 (0.18–0.43)	0.85 (0.75–0.94)	0.67 (0.48–0.85)	0.56 (0.45–0.67)	0.42 (0.27–0.54)

Note: Abbreviations: Trans-DLF, Transformer-based deep learning framework; 3D-CNN, 3D convolutional neural network; AUC, area under the curve; ACC, accuracy; SEN, sensitivity; SPE, specificity; PPV, positive prediction value; NPV, negative prediction value; CI, confidence interval; ITC, internal testing cohort; ETC1, external testing cohort 1; ETC2, external testing cohort 2

The AUC of the 3D-CNN baseline was numerically lower than that of the trans-DLF model, though this difference was not statistically significant, with AUCs ranging from 0.82 to 0.95. In contrast, the clinical model demonstrated markedly inferior discriminative capability, with AUCs ranging from 0.65 to 0.76 across all cohorts (all *p* < 0.001 vs. the trans-DLF) according to the DeLong test results, underscoring the limitation of relying solely on conventional clinical variables. Furthermore, no statistically significant AUC differences were observed between the trans-DLF and the multimodal nomogram (all *p* > 0.05).

**Fig. 4:**
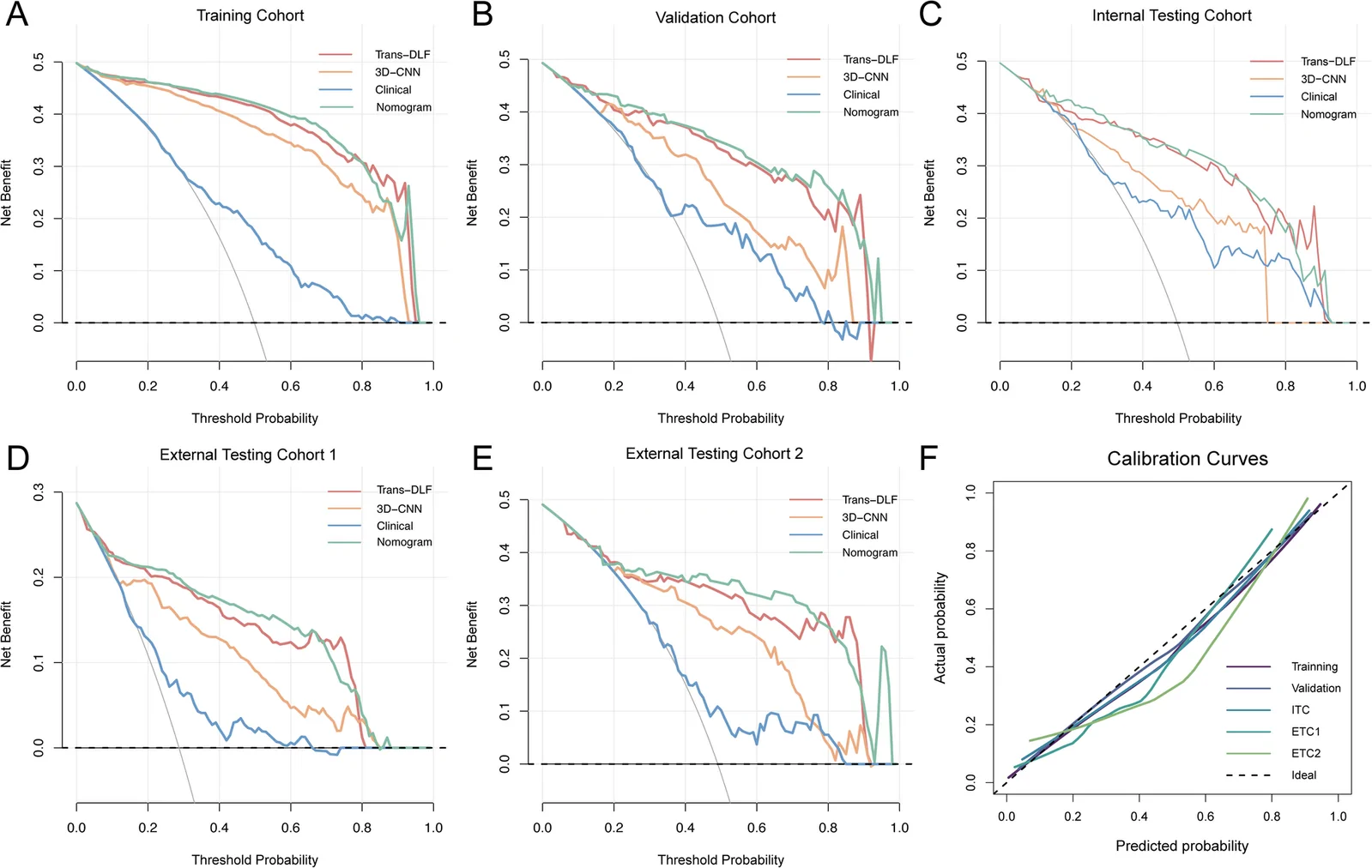
Clinical benefit and calibration performance of the transformer-based deep learning framework (trans-DLF), the 3D convolutional neural network (3D-CNN), the clinical model, and the multimodal nomogram. (**A**–**E**) Decision curve analyses illustrating the net clinical benefit of the trans-DLF, the 3D-CNN, and the clinical model across the training, validation, internal testing, and two external testing cohorts. (**F**) Calibration plots of the trans-DLF in all cohorts. The curves demonstrate strong agreement between predicted and observed probabilities of refractory *Mycoplasma pneumoniae* pneumonia (RMPP). The diagonal dashed line represents perfect calibration. Abbreviations: Trans-DLF, Transformer-based deep learning framework; 3D-CNN, 3D convolutional neural network; ITC, internal testing cohort; ETC1, external testing cohort 1; ETC2, external testing cohort 2

DCA suggested favorable clinical utility of the trans-DLF (Fig. 4A–E). Across a wide range of threshold probabilities, the trans-DLF provided a greater net benefit than both the 3D-CNN and clinical model, while maintaining a net benefit close to that of the multimodal nomogram. Additionally, the calibration curve analysis (Fig. 4F) showed good agreement between the predicted probabilities and observed outcomes.

Although hemoglobin was not retained in the multiple-imputation analysis, the performance of the clinical and multimodal models and the conclusions drawn from the AUC comparisons with the trans-DLF remained materially unchanged (Supplementary Tables S9–S10 and Supplementary Results 4.1).

### Subgroup analysis

**Fig. 5:**
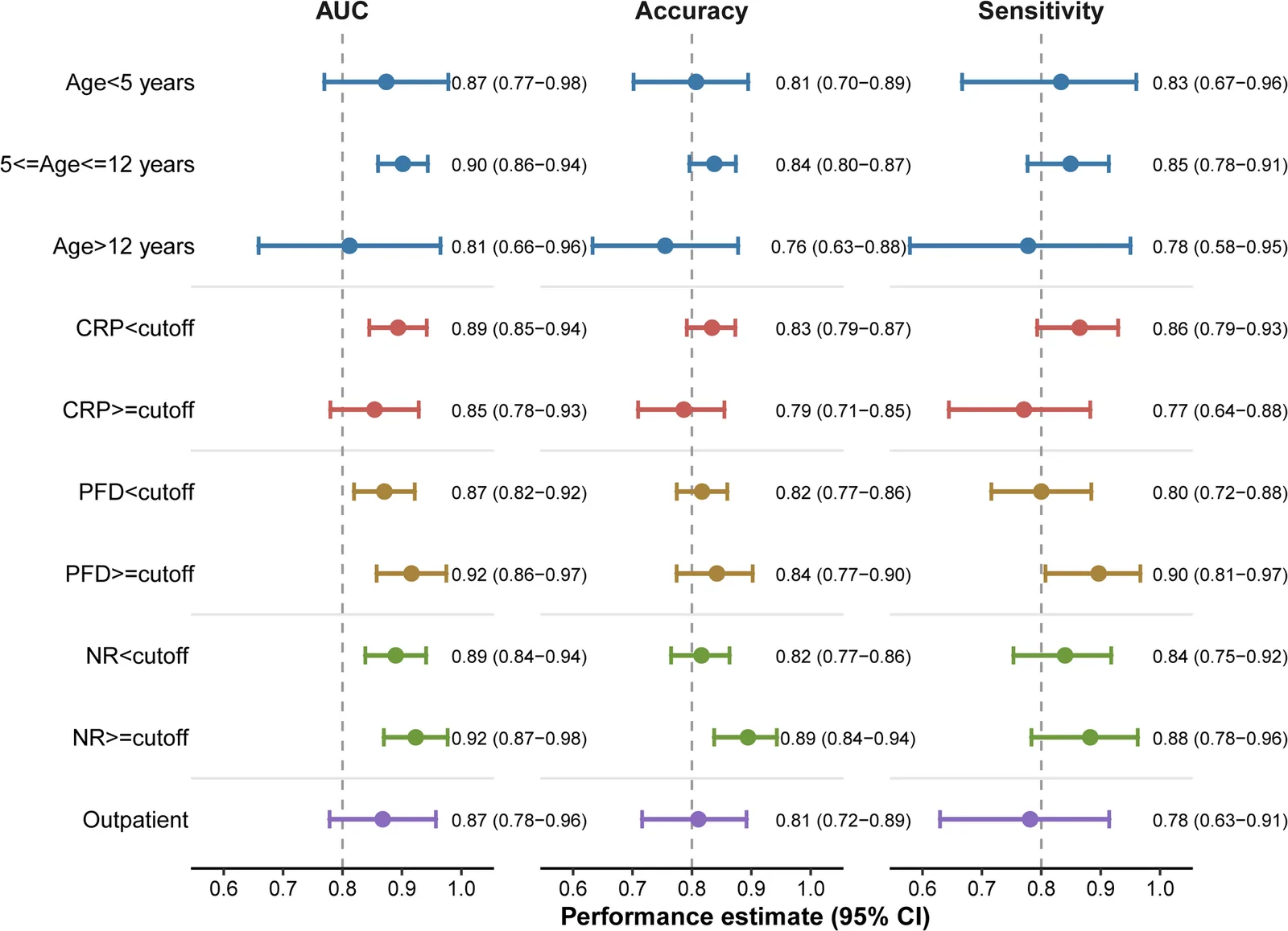
Subgroup analysis of the transformer-based deep learning framework (trans-DLF) in the combined external testing cohort. Forest plots illustrate the model’s performance across different clinical and demographic subgroups, measured by area under the curve (AUC), accuracy, and sensitivity. Cutoff values for pre-visit fever duration (PFD), C-reactive protein (CRP), and neutrophil ratio (NR) were 6.5 days, 18.44 mg/L, and 67.34%, respectively. Abbreviations: AUC, area under the curve; PFD, pre-visit fever duration; CRP, C-reactive protein; NR, neutrophil ratio; CI, confidence interval

To further evaluate the trans-DLF’s robustness, subgroup analyses of the combined external testing cohorts were performed across key clinical and demographic variables (Fig. 5 and Supplementary Table S11).

The trans-DLF achieved AUCs of 0.87 (95% CI, 0.77–0.98) for patients aged under 5 years, 0.90 (95% CI, 0.86–0.94) for those aged 5–12 years, and 0.81 (95% CI, 0.66–0.96) for those aged over 12 years. Furthermore, the model showed similar stability when stratified by clinical indicators, including PFD, CRP levels, and NR levels. Of particular clinical importance, the model retained a robust predictive ability in the outpatient subset (*n* = 74), achieving an AUC of 0.87 (95% CI, 0.78–0.96).

### Performance at Alternative Operating Thresholds

The two exploratory thresholds showed different transportability across cohorts (Supplementary Table S12). In the validation cohort, the sensitivity-oriented cutoff of 0.45 achieved a sensitivity of 0.91 (95% CI, 0.84–0.97). However, sensitivity decreased to 0.88, 0.87, and 0.83 in the internal and two external testing cohorts, respectively. In the outpatient subset, sensitivity was 0.78 (95% CI, 0.62–0.91), with 7 of 32 RMPP cases missed. In contrast, the specificity-oriented cutoff of 0.72 showed more consistent performance, maintaining a specificity of 0.89–0.90 across all independent testing cohorts and the outpatient subset, with positive predictive values of 0.74–0.87 and sensitivities of 0.72–0.78.

### Model interpretability

**Fig. 6:**
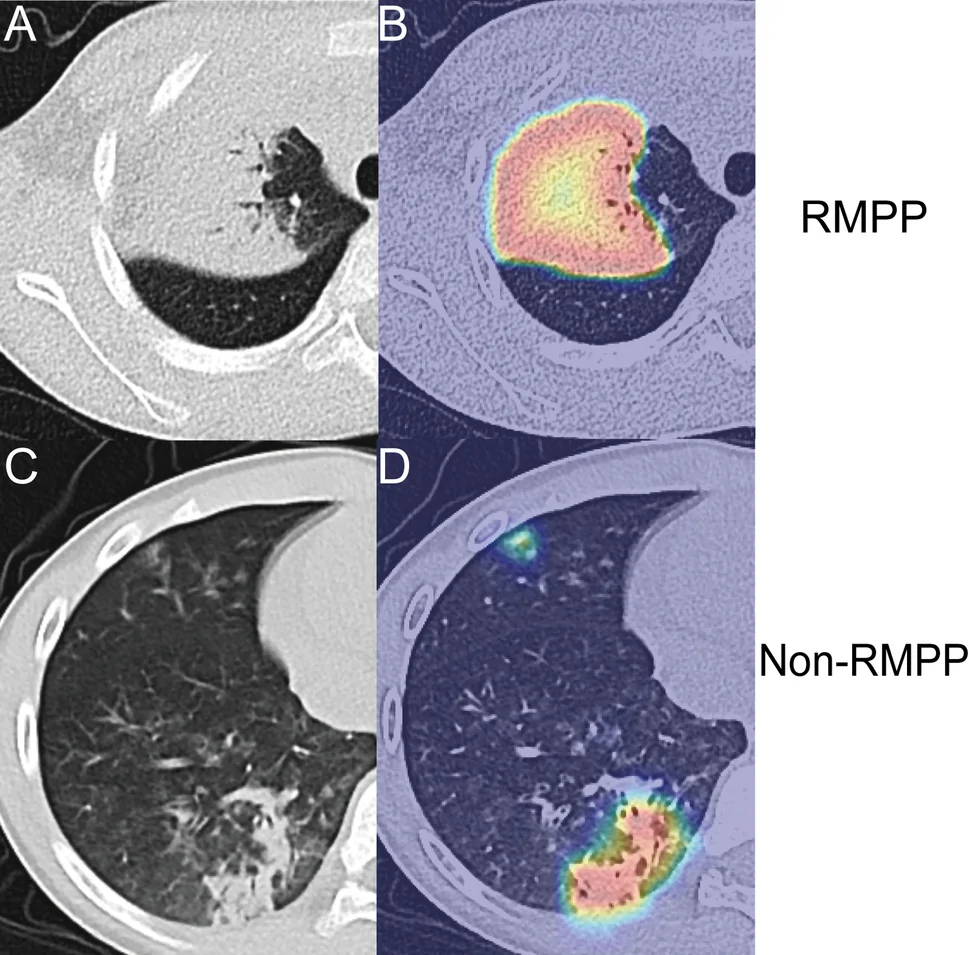
Representative CT images and corresponding gradient-weighted class activation mapping (Grad-CAM) visualizations illustrating the prediction interpretability of the transformer-based deep learning framework (trans-DLF). Each row, from left to right, shows the original axial CT image and the Grad-CAM heatmap overlaid on the CT image to highlight model attention. (**A**–**B**) In patients diagnosed with refractory *Mycoplasma pneumoniae* pneumonia (RMPP), Grad-CAM visualizations suggested broad and intense activations predominantly located in the right upper lobe, corresponding to extensive areas of consolidation. (**C**–**D**) In contrast, in non-RMPP patients who exhibited relatively smaller areas of consolidation alongside ground-glass opacities (GGOs), the activation maps appeared to focus primarily on the consolidated regions, with lower activation in the GGO areas. Abbreviations: RMPP, refractory *Mycoplasma pneumoniae* pneumonia

Grad-CAM was used to provide post hoc visualization of image regions associated with the predictions of the trans-DLF (Fig. 6). In RMPP cases, the model’s attention was concentrated on extensive consolidation, a well-recognized radiological marker for refractory disease. Conversely, in non-RMPP cases characterized by smaller consolidations and ground-glass opacities (GGOs), the model selectively focused on the consolidated areas while assigning significantly lower weights to GGO regions.

## Discussion

This study developed and validated a deep learning framework (trans-DLF) that leveraged only baseline chest CT data to automatically stratify the risk of RMPP in children. The model exhibited consistent and robust discriminative performance across multiple cohorts, including two independent external testing sets and clinically relevant subgroups such as outpatients. Notably, despite relying exclusively on imaging data, the trans-DLF showed no statistically significant AUC difference from the multimodal nomogram and yielded a higher net benefit than the 3D-CNN across most threshold probabilities in DCA. Collectively, these findings support the trans-DLF as a clinically promising CT-only tool for RMPP risk stratification in pediatric MPP.

The post-imaging phase represents a critical decision-making window during which clinicians must promptly determine whether to initiate corticosteroids or escalate antibiotic therapy. While current multimodal models are often constrained by their dependence on time-intensive laboratory data, manual entry, or complex image preprocessing [10, 27, 30, 37, 40], the CT-based unimodal model provides rapid, automated risk stratification from existing scans without requiring additional testing. However, the model is intended for patients who have already undergone clinically indicated CT rather than for population screening or early-stage prediction. Because CT was generally performed in patients with clinical non-improvement or suspected severe pulmonary involvement, the cohort was enriched for higher-risk cases, with an RMPP prevalence of 43.95%, and imaging was obtained a median of 4 days after the initiation of macrolide therapy. Accordingly, the model should be interpreted as a short-term post-CT risk stratification tool, and its PPV, net clinical benefit, and generalizability should be considered within this selected population until further validated in earlier-stage, lower-prevalence, or unselected MPP cohorts.

To ensure the robustness and generalizability of our findings, a multi-center study design was employed, encompassing a national referral center and two independent external institutions. This approach was specifically intended to enhance population diversity and capture a broad spectrum of clinical settings within the hierarchical healthcare system. Across these diverse settings, the trans-DLF demonstrated high discriminative ability, with AUCs of 0.90 in internal validation and 0.89 across two independent external cohorts. Its performance remained stable in pragmatic outpatient and emergency scenarios (AUC = 0.87). Although a slight decrease was noted in the small subgroup of patients over 12 years old (AUC = 0.81), the wide 95% CI (0.66–0.96) suggests this is likely due to the limited sample size rather than an intrinsic algorithmic deficit. Overall, these findings support the transportability of model discrimination across institutions, care settings, and patient subgroups.

However, consistent discrimination across cohorts did not guarantee stable threshold-dependent performance. All three operating thresholds, including the primary Youden-derived threshold and the two exploratory thresholds, were determined exclusively in the validation cohort and applied unchanged to all testing cohorts, without adjustment for cohort-specific RMPP prevalence or recalibration of predicted probabilities. This design minimized optimistic bias from test-set optimization and enabled a direct assessment of threshold transportability. It also demonstrated the expected effect of outcome prevalence on predictive values. Because external testing cohort 1 had a lower RMPP prevalence than the validation cohort and external testing cohort 2, application of the same thresholds yielded a lower PPV and a higher NPV. The exploratory thresholds also showed asymmetric transportability. The sensitivity-oriented cutoff of 0.45 met the prespecified target in the validation cohort but achieved a sensitivity of only 0.78 in the outpatient subset, limiting its suitability for stand-alone triage or exclusion of RMPP. By contrast, the specificity-oriented cutoff of 0.72 maintained a specificity of 0.89–0.90 across the independent testing cohorts and may serve as a conservative high-risk signal. Prospective implementation should therefore include setting-specific calibration and independent validation of thresholds selected according to the intended clinical use and the relative consequences of false-positive and false-negative classifications.

From a methodological perspective, the trans-DLF offers a plausible framework for modeling diffuse pneumonia by using self-attention to capture global contextual relationships and interslice dependencies across the entire lung volume [41, 42]. Compared with traditional 3D-CNNs, which primarily emphasize local spatial features, the transformer-based architecture may be useful for characterizing the distribution and extent of pulmonary abnormalities, both of which are clinically relevant in RMPP. In the present study, the AUC differences between the trans-DLF and 3D-CNN were not statistically significant; therefore, the present findings do not establish definitive architectural superiority. However, DCA showed that the trans-DLF yielded a higher net benefit than the 3D-CNN across most evaluated threshold probabilities, suggesting potential value for clinical decision support. Furthermore, despite its streamlined unimodal design, the trans-DLF showed no statistically significant AUC difference from the multimodal nomogram. These findings suggest that the transformer architecture may capture high-dimensional imaging features associated with systemic inflammation and tissue damage. Therefore, CT-only transformer-based modeling may offer practical value for post-CT risk stratification by providing timely risk estimates from a single imaging source when timely clinical decision support may be beneficial.

To improve model interpretability, Grad-CAM was used to visualize image regions associated with model predictions. The resulting activation maps suggested that predictions were influenced by pulmonary lesions rather than predominantly by background structures. In particular, stronger activation was observed in regions of consolidation, a recognized imaging feature associated with RMPP, whereas weaker activation was observed in areas of nonspecific GGO [23, 25]. This agreement with established radiologic knowledge provides supportive evidence for the clinical plausibility of the model predictions.

Despite these promising results, several limitations should be acknowledged. First, because the study included only children who underwent clinically indicated CT and CT was obtained relatively close to the minimum treatment duration required for formal RMPP diagnosis, the applicability of the model to unselected, lower-risk, or earlier-stage MPP populations remains uncertain and requires prospective validation. Second, although Grad-CAM heatmaps provide valuable visual explanations, they are a post hoc interpretation technique; more comprehensive mapping between the learned features and a broader spectrum of radiologic semantics would further strengthen explainability. Third, despite multi-center validation, prospective studies in varied healthcare settings and diverse ethnic populations are needed to fully establish the model’s generalizability. In addition, the reference clinical model was developed using univariable pre-screening, which may have excluded predictors that became informative only after adjustment and thereby introduced selection bias. Finally, the clinical utility of the trans-DLF, including its effects on workflow efficiency, decision-making, and patient outcomes, requires prospective evaluation. The contributions of individual preprocessing steps also remain to be established through systematic ablation analyses.

## Conclusions

In summary, the trans-DLF was developed and validated as a rapid, automated research prototype for post-CT risk stratification of RMPP in children undergoing clinically indicated chest CT. By using CT data alone, the model enables timely risk assessment without requiring additional laboratory inputs and may support subsequent clinical reassessment and management planning. The trans-DLF may therefore streamline imaging-based risk stratification in acute care settings. However, it is not intended for population-level screening, and its applicability to unselected or lower-risk MPP populations requires prospective validation.

## Supplementary Information

Below is the link to the electronic supplementary material.

Supplementary Material 1

## Data Availability

The datasets generated and/or analyzed during the current study are not publicly available due to ethical and privacy considerations, but are available from the corresponding author on reasonable request.

## **Abbreviations**

MPP: Mycoplasma pneumoniae pneumonia
RMPP: Refractory mycoplasma pneumoniae pneumonia
CT: Computed tomography
PFD: Pre-visit fever duration
CRP: C-reactive protein
NR: Neutrophil ratio
DL: Deep learning
Trans-DLF: Transformer-based deep learning framework
3D-CNN: Three-dimensional convolutional neural network
AUC: Area under the receiver operating characteristic curve
DCA: Decision curve analysis
