# Supplementary material for: A CT-based deep learning model for the automated risk stratification of refractory Mycoplasma pneumoniae pneumonia in children
Source: BMC Med Imaging. 2026 Jul 17;26:521. doi: 10.1186/s12880-026-02590-y (PMC13660281; doi:10.1186/s12880-026-02590-y)
Supplement: Supplementary file 1 [file 12880_2026_2590_MOESM1_ESM.docx]

**Supplementary Data**

[**1**	**Supplementary Materials**	1](#_Toc234061423)

[**1.1**	**Diagnostic Criteria for *Mycoplasma pneumoniae* Pneumonia (*MPP)* [1]**	1](#_Toc234061424)

[**1.2**	**Classification of MPP Severity**	1](#_Toc234061425)

[**1.3**	**Development of the Deep Learning-based Models**	1](#_Toc234061426)

[**1.4**	**Multiple-Imputation Sensitivity Analysis**	3](#_Toc234061427)

[**2**	**Supplementary Tables**	5](#_Toc234061428)

[**2.1**	**Table S1. Summary of CT Acquisition Parameters in the Three Participating Centers.**	5](#_Toc234061429)

[**2.2**	**Table S2. Summary of Variables Used in the Study and their Corresponding Missing Proportions.**	6](#_Toc234061430)

[**2.3**	**Table S3. Completed CLAIRE Reporting Checklist for the Trans-DLF Study.**	8](#_Toc234061431)

[**2.4**	**Table S4. Laboratory and Radiological Characteristics of Patients with Refractory and Non-Refractory *Mycoplasma Pneumonia* Pneumonia in Center 1.**	10](#_Toc234061432)

[**2.5**	**Table S5. Characteristics of Patients with Refractory and Non-Refractory *Mycoplasma Pneumonia* Pneumonia in the External Testing Cohort.**	12](#_Toc234061433)

[**2.6**	**Table S6. Pooled Univariable and Multivariable Logistic Regression Analyses of Clinical Predictors for RMPP in the Training Cohort Across 20 Multiply Imputed Datasets.**	14](#_Toc234061434)

[**2.7**	**Table S7. Predictive Performance of the Multimodal Nomogram.**	15](#_Toc234061435)

[**2.8**	**Table S8. Absolute Classification Counts for the Predictive Models Across the Study Cohorts.**	16](#_Toc234061436)

[**2.9**	**Table S9. Pooled Performance of the Clinical and Multimodal Models Across 20 Multiply Imputed Datasets.**	17](#_Toc234061437)

[**2.10**	**Table S10. Pooled DeLong Test Results for AUC Comparisons Across 20 Multiply Imputed Datasets.**	19](#_Toc234061438)

[**2.11**	**Table S11. The prediction performance of Trans-DLF in the Subgroup Analysis.**	20](#_Toc234061439)

[**2.12**	**Table S12. Performance of the trans-DLF at clinically oriented operating thresholds.**	23](#_Toc234061440)

[**3**	**Supplementary Figure**	25](#_Toc234061441)

[**3.1**	**Figure S1. Distribution of Patient Age in the Center 1.**	25](#_Toc234061442)

[**3.2**	**Figure S2. Multimodal nomogram for predicting refractory *Mycoplasma pneumoniae* pneumonia (RMPP).**	25](#_Toc234061443)

[**3.3**	**Figure S3. Multimodal Nomogram Constructed Using Pooled Regression Coefficients Across 20 Multiply Imputed Datasets.**	26](#_Toc234061444)

[**3.4**	**Figure S4. Predictive model performance for refractory *Mycoplasma pneumoniae* pneumonia (RMPP) in Training Cohort.**	26](#_Toc234061445)

[**4**	**Supplementary Result**	28](#_Toc234061447)

[**4.1**	**Results of the Multiple-Imputation Sensitivity Analysis**	28](#_Toc234061448)

# 1 **Supplementary Materials**

# 1.1 **Diagnostic Criteria for *Mycoplasma pneumoniae* Pneumonia (*MPP)* [1]**

A diagnosis of MPP required both clinical suspicions supported by radiographic findings and definitive laboratory confirmation:

1) **Clinical and Radiographic Evidence**: A clinical presentation including fever, cough, or auscultatory abnormalities (e.g., crackles, wheezing), combined with imaging evidence of pulmonary infiltrates.
2) **Laboratory Confirmation**: Required fulfillment of at least one of the following: (a) a positive polymerase chain reaction (PCR) test for *M. pneumoniae* DNA or RNA from nasopharyngeal secretions; (b) a ≥4-fold rise in *M. pneumoniae*-specific IgG antibody titers in paired serum samples; or (c) a single IgM antibody titer of 1:160 or higher.

# 1.2 **Classification of MPP Severity**

Following diagnosis, MPP severity was categorized into three levels based on the following criteria:

1) **Mild MPP:** Cases not meeting the definitions for severe or critical MPP.
2) **Severe MPP:** A case was classified as severe if one or more of the following conditions were met [1]:
   a) A sustained high fever (≥39°C) for ≥5 days, or a febrile period lasting ≥7 days without a descending peak temperature.
   b) The onset of wheezing, dyspnea, respiratory distress, chest pain, or hemoptysis.
   c) Manifestation of extrapulmonary complications not classified as critical illness.
   d) Resting oxygen saturation (SpO_2_) of 93% or less on room air.
   e) Chest imaging demonstrating one or more of the following:
      i. Dense consolidation involving ≥2/3 of one lung lobe or present in at least two lobes (regardless of size), with or without moderate-to-large pleural effusion.
      ii. Diffuse involvement of a single lung or bronchiolitis in ≥4/5 of both lungs, with potential mucus plug formation and subsequent atelectasis.
   f) Radiographic disease progression of more than 50% within 24–48 hours alongside clinical worsening.
   g) A pronounced increase in at least one key biomarker: C-reactive protein (CRP), lactate dehydrogenase (LDH), or D-dimer.
3) **Critical MPP:** Characterized by respiratory failure and/or the presence of life-threatening extrapulmonary complications that require mechanical ventilation or other forms of life support.

# 1.3 **Development of the Deep Learning-based Models**

1) **Model Architecture**

We developed a Transformer-based Deep Learning Framework (Trans-DLF) to predict refractory *Mycoplasma pneumoniae* pneumonia (RMPP) from pediatric chest CT scans. The model was designed to capture global inter-slice dependencies and spatial-contextual relationships across the entire lung volume.

2) **Lung Segmentation and Preprocessing**

All CT volumes were first processed using a lung segmentation model to obtain lung masks, which were subsequently used to define a tight bounding box enclosing the lungs for region-of-interest (ROI) cropping. The segmentation model achieved a Dice coefficient of 0.975 for whole-lung segmentation, indicating reliable and stable delineation of lung regions [2]. After lung-based cropping, each CT volume was resampled to a fixed input size of 192 × 256 × 256 voxels to ensure spatial consistency across all cases. The CT images were displayed using a lung window with a window level of −600 HU and a window width of 1500 HU, corresponding to an intensity range of −1350 to 150 HU. Voxel intensities outside this range were clipped to the respective lower or upper limits and subsequently linearly scaled to the range of 0 to 1. The intensity-scaled volume was then standardized on a per-patient basis using Z-score normalization based on its own mean and standard deviation. This standardized preprocessing pipeline was applied consistently to the training, validation, internal testing, and external testing cohorts.

3) **Data Augmentation**

Data augmentation was applied exclusively to the training cohort and was performed during model training. The augmentation pipeline included random rotations with angles uniformly sampled between −15° and 15°, random flipping along the spatial axes, additive Gaussian noise with a mean of 0 and a standard deviation of 0.05. Each augmentation operation was applied with an approximate probability of 0.5. No data augmentation was applied to the validation, internal testing, or external testing cohorts.

4) **Slice Grouping and Feature Encoding**

To efficiently model inter-slice continuity while maintaining manageable computational cost, every three consecutive slices were grouped as one unit. Each 3-slice input (1×3×256×256) was fed into a ResNet-18 backbone, pretrained on ImageNet and fine-tuned exclusively on the training cohort for 2D feature extraction. This process generated a sequence of 64 feature embeddings, each representing local morphological and textural characteristics of the lung.

5) **Transformer-based Feature Modeling**

The core of our architecture is a slice-level Transformer encoder designed to model long-range contextual dependencies. For each of the 64 encoded slice groups, spatial features were globally aggregated into [1×256] vectors using max pooling. This sequence of embeddings was then processed by a six-layer Transformer encoder. The encoder utilized multi-head self-attention (MHSA) modules to capture non-local inter-slice dependencies, relative positional encoding (RelPE) to preserve spatial order along the z-axis, and feed-forward networks with residual connections for feature refinement.

This attention-based mechanism enabled the model to dynamically learn interactions between disparate lung regions and understand how distributed inflammatory patterns contribute to RMPP development. Finally, the output from the Transformer was passed to a multilayer perceptron (MLP) classifier to yield a probabilistic RMPP risk prediction.

6) **Model Implementation, Hyperparameters, and Training Strategy**

The proposed model was implemented in PyTorch and optimized using the cross-entropy loss function with the Adam optimizer. A weight decay of 1 × 10⁻⁵ was applied for regularization. A module-specific learning rate strategy was adopted, with a learning rate of 1 × 10⁻⁶ assigned to the Transformer encoder and 1 × 10⁻⁴ for all other trainable parameters, enabling stable optimization of pretrained components while maintaining sufficient learning capacity in task-specific layers. The transformer encoder was configured with an embedding dimension of 256, eight attention heads, a feed-forward dimension of 512, and a dropout rate of 0.1. Xavier initialization was used for weight initialization. To improve training stability, gradient clipping with a maximum norm of 1.0 was applied. The model was trained for a maximum of 100 epochs with a batch size of two per GPU. A cosine annealing learning rate schedule was adopted to gradually reduce the learning rate during training. Early stopping was employed based on validation performance to prevent overfitting. The training cohort was used exclusively for parameter optimization, while the validation cohort was used for model selection and hyperparameter tuning. The checkpoint achieving the highest validation accuracy was retained as the final model. Performance evaluation was conducted solely on the internal and external testing cohorts, which were not involved in any stage of training or model selection. To ensure reproducibility, all experiments were conducted with fixed random seeds.

7) **Comparative Model: 3D Convolutional Baseline**

To validate the effectiveness of the Transformer’s long-range dependency modeling, we implemented a 3D convolutional neural network (3D-CNN) as a baseline. In this model, all slice features were stacked along the channel dimension and fed into 3D convolutional layers to jointly learn spatial-channel representations. Unlike the Transformer, which explicitly models inter-slice relationships through self-attention, the 3D-CNN captures only local volumetric correlations.

# 1.4 **Multiple-Imputation Sensitivity Analysis**

Missing values in eligible variables were addressed using multiple imputation by chained equations with predictive mean matching. Twenty completed datasets were generated separately within each cohort to prevent information leakage between cohorts. Variable selection was performed exclusively in the training cohort. Univariable logistic regression was conducted for each candidate predictor in each imputed dataset, and the resulting coefficient estimates and standard errors were pooled across imputations using Rubin rules [3]. Predictors with a pooled *P* value of less than 0.05 were entered into the multivariable logistic regression analysis. The multivariable model was fitted separately in each imputed dataset, and predictors that remained statistically significant after pooling were retained in the multiple-imputation clinical model.

For each imputation, the clinical and multimodal models were fitted in the corresponding imputed training dataset. The multimodal model included the predictors retained in the clinical model together with the trans-DLF probability. Regression coefficients were pooled across imputations using Rubin rules, and the pooled coefficients of the multimodal model were used to construct the corresponding nomogram.

Model discrimination was assessed primarily using the area under the receiver operating characteristic curve (AUC). Predictions and performance metrics for the clinical and multimodal models were calculated separately within each imputation. Patient-level predicted probabilities were averaged across imputations for threshold selection. For each model, the threshold that maximized the Youden index in the validation cohort was fixed and subsequently applied to the training, internal testing, and external testing cohorts.

Within each imputation, the AUC, accuracy, sensitivity, specificity, positive predictive value, and negative predictive value were calculated. Stratified bootstrap resampling with 2,000 replicates was used to estimate the within-imputation variance of each performance metric. The resulting estimates were pooled across imputations using Rubin rules. Metrics bounded between 0 and 1 were pooled on the logit scale and subsequently back-transformed, thereby accounting for both sampling variability and between-imputation uncertainty.

Within each cohort, the AUC of the trans-DLF was compared with those of the clinical model, the 3D-CNN, and the multimodal nomogram. For comparisons involving the clinical model or multimodal nomogram, the AUC difference and corresponding DeLong variance were estimated separately within each imputation and subsequently pooled across imputations using Rubin rules.

**Reference**

1.	National Health Commission of the People’s Republic of China (2023) Guidelines for the diagnosis and treatment of Mycoplasma pneumoniae pneumonia in children (2023 edition). Inter J Epidemiol Infect Dis 50:79–85. https://doi.org/10.3760/cma.j.cn331340-20230217-00023

2.	Bai Y, Wang X, Zhou Z, et al (2023) Pulmonary segments segmentation with hierarchical weak labels. In: 2023 IEEE 20th International Symposium on Biomedical Imaging (ISBI). IEEE, pp 1–5

3.	(1987) Randomization-Based Evaluations. In: Multiple Imputation for Nonresponse in Surveys. John Wiley & Sons, Ltd, pp 113–153

# 2 **Supplementary Tables**

# 2.1 **Table S1. Summary of CT Acquisition Parameters in the Three Participating Centers.**

| Center | Scanning equipment | Tube voltage (kV) | Tube current (mAs) | Pitch | Slice thickness (mm) |
| --- | --- | --- | --- | --- | --- |
| Center 1 | SOMATOM Force | 70, 80, 90, 100 | AEC | 0.9, 1.0, 1.4, 1.9 | 0.6, 1 |
|  | Optima CT660 | 80, 100, 120 |  | 0.96875, 0.984375, 1.375 | 0.625, 1.25 |
| Center 2 | SOMATOM Definition Flash | 80, 100, 120 | AEC | 1.2, 1.4, 1.5, 2.85, 3 | 0.6, 1, 1.5 |
|  | SOMATOM Force | 70, 80, 90, 100, 110, 120 |  | 1.2, 1.5, 1.9, 2 |  |
| Center 3 | IQon - Spectral CT | 120 | AEC | 1.258 | 0.625, 1 |
|  | SOMATOM Definition Flash | 100, 120 |  | 1.2 | 0.6, 1 |
|  | SOMATOM Force | 80, 90, 100, 110 |  | 0.6, 1.2, 1.4, 2 | 0.6, 1 |

Abbreviations: AEC, automatic exposure control.

# 2.2 **Table S2. Summary of Variables Used in the Study and their Corresponding Missing Proportions.**

|  | Training Cohort | Validation Cohort | Internal Testing Cohort | External Testing Cohort 1 | External Testing Cohort 2 (Inpatient) | External Testing Cohort 2 (Outpatient) | Included in Final Analysis |
| --- | --- | --- | --- | --- | --- | --- | --- |
| Number of Patients | 506 | 140 | 139 | 331 | 34 | 74 |  |
| Sex | 0 (0.0%) | 0 (0.0%) | 0 (0.0%) | 0 (0.0%) | 0 (0.0%) | 0 (0.0%) | Yes |
| Age | 0 (0.0%) | 0 (0.0%) | 0 (0.0%) | 0 (0.0%) | 0 (0.0%) | 0 (0.0%) | Yes |
| Comorbidity | 0 (0.0%) | 0 (0.0%) | 0 (0.0%) | 0 (0.0%) | 0 (0.0%) | 0 (0.0%) | Yes |
| PFD | 0 (0.0%) | 0 (0.0%) | 0 (0.0%) | 0 (0.0%) | 0 (0.0%) | 0 (0.0%) | Yes |
| PCD | 0 (0.0%) | 0 (0.0%) | 0 (0.0%) | 0 (0.0%) | 0 (0.0%) | 0 (0.0%) | Yes |
| Severe/Critical Pneumonia | 0 (0.0%) | 0 (0.0%) | 0 (0.0%) | 0 (0.0%) | 0 (0.0%) | 0 (0.0%) | Yes |
| ROS | 0 (0.0%) | 0 (0.0%) | 0 (0.0%) | 0 (0.0%) | 0 (0.0%) | 0 (0.0%) | Yes |
| T | 41 (8.1%) | 15 (10.7%) | 10 (7.2%) | 8 (2.4%) | 0 (0.0%) | 12 (16.2%) | Yes |
| P | 41 (8.1%) | 15 (10.7%) | 10 (7.2%) | 8 (2.4%) | 0 (0.0%) | 12 (16.2%) | Yes |
| R | 41 (8.1%) | 15 (10.7%) | 10 (7.2%) | 8 (2.4%) | 0 (0.0%) | 12 (16.2%) | Yes |
| SpO_2_ | 214 (42.3%) | 64 (45.7%) | 54 (38.8%) | 35 (10.6%) | 0 (0.0%) | 12 (16.2%) | No |
| CRP | 24 (4.7%) | 4 (2.9%) | 6 (4.3%) | 12 (3.6%) | 0 (0.0%) | 2 (2.7%) | Yes |
| ESR | 45 (8.9%) | 9 (6.4%) | 21 (15.1%) | 173 (52.3%) | 13 (38.2%) | 74 (100.0%) | No |
| PCT | 35 (6.9%) | 6 (4.3%) | 7 (5.0%) | 127 (38.4%) | 8 (23.5%) | 66 (89.2%) | No |
| WBC | 24 (4.7%) | 4 (2.9%) | 6 (4.3%) | 82 (24.8%) | 0 (0.0%) | 0 (0.0%) | Yes |
| NR | 24 (4.7%) | 4 (2.9%) | 6 (4.3%) | 82 (24.8%) | 0 (0.0%) | 0 (0.0%) | Yes |
| MR | 24 (4.7%) | 4 (2.9%) | 6 (4.3%) | 94 (28.4%) | 0 (0.0%) | 0 (0.0%) | Yes |
| LR | 24 (4.7%) | 4 (2.9%) | 6 (4.3%) | 82 (24.8%) | 0 (0.0%) | 0 (0.0%) | Yes |
| BR | 24 (4.7%) | 4 (2.9%) | 6 (4.3%) | 82 (24.8%) | 0 (0.0%) | 0 (0.0%) | Yes |
| ER | 24 (4.7%) | 4 (2.9%) | 6 (4.3%) | 82 (24.8%) | 0 (0.0%) | 0 (0.0%) | Yes |
| HGB | 24 (4.7%) | 4 (2.9%) | 6 (4.3%) | 82 (24.8%) | 0 (0.0%) | 0 (0.0%) | Yes |
| PLT | 24 (4.7%) | 4 (2.9%) | 6 (4.3%) | 82 (24.8%) | 0 (0.0%) | 0 (0.0%) | Yes |
| D-dimer | 33 (6.5%) | 5 (3.6%) | 13 (9.4%) | 177 (53.5%) | 0 (0.0%) | 72 (97.3%) | No |
| LDH | 5 (1.0%) | 0 (0.0%) | 0 (0.0%) | 246 (74.3%) | 0 (0.0%) | 69 (93.2%) | No |
| Atelectasis | 0 (0.0%) | 0 (0.0%) | 0 (0.0%) | 0 (0.0%) | 0 (0.0%) | 0 (0.0%) | Yes |
| Pleural effusion | 0 (0.0%) | 0 (0.0%) | 0 (0.0%) | 0 (0.0%) | 0 (0.0%) | 0 (0.0%) | Yes |

Note: Numbers are presented as counts with percentages in parentheses, calculated relative to the total number of patients in each cohort. And the last column indicates whether the variable was retained for subsequent model development and analysis.

Abbreviations: PFD, pre-visit fever duration; PCD, pre-visit cough duration; ROS, requirement for oxygen support; T, temperature; P, pulse rate; R, respiratory rate; SpO₂, peripheral capillary oxygen saturation; CRP, C-reactive protein; ESR, erythrocyte sedimentation rate; PCT, procalcitonin; WBC, white blood cell count; NR, neutrophil ratio; MR, monocyte ratio; LR, lymphocyte ratio; BR, basophil ratio; ER, eosinophil ratio; HGB, hemoglobin; PLT, platelet count; LDH, lactate dehydrogenase.

# 2.3 **Table S3. Completed CLAIRE Reporting Checklist for the Trans-DLF Study.**

| **SECTION** | **ITEM NO.** | **ITEM** | **REPORTED* (Yes / No / N/A)** | **REPORTED SECTION** |
| --- | --- | --- | --- | --- |
| Title | 01 | Definition of the clinical scope of the model | Yes | Title; Abstract (Background and Methods). |
| Introduction | 02 | Objective of the proposed model | Yes | Abstract (Background); Background, paragraphs 2–5. |
| Materials and Methods | 03 | Ethical considerations | Yes | Methods, opening paragraph; Declarations (Ethics approval and consent to participate; Availability of data and materials) |
| Materials and Methods | 04 | Characterisation of primary data | Yes | Methods 2.1–2.2; Table 1; Figure 2; Supplementary Materials 1.1–1.2; Supplementary Tables S1, S2, S4, and S5. |
| Materials and Methods | 05 | Characterisation of secondary data | N/A | No secondary clinical or synthetic datasets were used. ImageNet-pretrained weights were used only for initialization and are considered under Item 08. |
| Materials and Methods | 06 | Eligibility criteria | Yes | Methods 2.1, 2.2, and 2.4; Supplementary Table S2; Supplementary Material 1.4; Discussion, limitations paragraph. |
| Materials and Methods | 07 | Pre-processing | Yes | Methods 2.1 and 2.3; Figure 1; Supplementary Material 1.3. |
| Materials and Methods | 08 | Model description | Yes | Methods 2.3; Supplementary Material 1.3. |
| Materials and Methods | 09 | Validation | Yes | Methods 2.1 and 2.5; Supplementary Material 1.3. |
| Results | 10 | Performance metrics | No | Results 3.2; Table 3; Supplementary Tables S7–S10 and S12. Performance metrics are reported for the final model, but performance without data augmentation was not evaluated. |
| Results | 10 | Absolute values | Yes | Table 3 and Supplementary table S8. |
| Results | 10 | Confidence intervals | Yes | Methods 2.5; Results 3.2–3.3; Table 3; Supplementary Tables S7-S12. |
| Results | 11 | Visual representation | Yes | Figures 3–6 and S2–S4, including ROC curves, waterfall plots, calibration plots, DCA, subgroup plots, and Grad-CAM visualizations. |
| Discussion | 12 | Clinical applicability | No | Discussion, paragraphs 2 and 7. Workflow integration, effects on interpretation time and clinical workload, and the individual contributions of preprocessing steps were not directly evaluated. |
| Discussion | 13 | External validation and generalisability | Yes | Results 3.2–3.3; Discussion, external-validation and generalizability paragraphs; Supplementary Table S1. |
| Discussion | 14 | Limitations and opportunities for improvement | Yes | Discussion, final paragraph. |
| Conclusion | 15 | Final synthesis | Yes | Conclusions. |

Note: Each item was classified as “Yes,” “No,” or “N/A” according to the CLAIRE framework. A justification is provided for each item classified as “No” or “N/A.” Abbreviations: CLAIRE, Completeness, Learnability, Applicability, Interpretability, Reproducibility, and Evaluation; N/A, not applicable.

# 2.4 **Table S****4. Laboratory and Radiological Characteristics of Patients with Refractory and Non-Refractory *Mycoplasma Pneumonia* Pneumonia in Center 1.**

| Variables | Training Cohort (n = 506) | | | Validation Cohort (n = 140) | | | Internal Testing Cohort (n = 139) | | |
| --- | --- | --- | --- | --- | --- | --- | --- | --- | --- |
|  | Non-RMPP (n = 254) | RMPP (n = 252) | *p*-value | Non-RMPP (n = 71) | RMPP (n = 69) | *p*-value | Non-RMPP (n = 70) | RMPP (n = 69) | *p*-value |
| CRP, mg/L (median [IQR]) | 9.38 [4.21, 17.47] | 12.69 [5.37, 24.74] | 0.02* | 10.32 [4.47, 18.40] | 10.44 [3.70, 29.19] | 0.70 | 8.32 [2.74, 21.47] | 10.24 [5.11, 21.07] | 0.37 |
| WBC, ×10^9^/L (median [IQR]) | 7.00 [5.55, 8.70] | 7.32 [5.79, 10.34] | 0.04* | 6.78 [5.65, 8.18] | 7.96 [5.90, 9.68] | 0.04* | 7.34 [5.71, 8.80] | 6.53 [5.28, 9.45] | 0.48 |
| NR, % (median [IQR]) | 64.93 [55.43, 71.06] | 65.98 [57.86, 72.24] | 0.08 | 62.71 [54.89, 70.61] | 67.66 [57.39, 74.14] | 0.13 | 62.59 [52.44, 71.69] | 64.34 [58.18, 71.50] | 0.51 |
| MR, % (median [IQR]) | 7.02 [5.48, 8.60] | 7.04 [5.32, 8.49] | 0.65 | 6.81 [5.09, 8.08] | 7.13 [5.18, 8.34] | 0.49 | 7.18 [5.19, 8.57] | 7.45 [5.45, 8.98] | 0.80 |
| LR, % (median [IQR]) | 25.98 [19.76, 35.34] | 24.63 [18.84, 33.18] | 0.07 | 27.75 [20.67, 36.48] | 24.15 [18.16, 32.77] | 0.10 | 27.52 [20.14, 34.93] | 26.47 [19.02, 32.52] | 0.38 |
| BR, % (median [IQR]) | 0.16 [0.10, 0.26] | 0.17 [0.09, 0.28] | 0.51 | 0.15 [0.09, 0.20] | 0.16 [0.04, 0.23] | 0.88 | 0.16 [0.12, 0.28] | 0.16 [0.00, 0.24] | 0.27 |
| ER, % (median [IQR]) | 0.73 [0.21, 2.21] | 0.60 [0.15, 2.15] | 0.18 | 0.68 [0.15, 2.23] | 0.54 [0.12, 1.55] | 0.45 | 0.59 [0.18, 1.64] | 0.97 [0.29, 2.18] | 0.12 |
| HGB, g/L (median [IQR]) | 123.00 [117.00, 129.00] | 121.50 [115.00, 127.00] | 0.01* | 120.50 [114.50, 128.25] | 120.00 [114.00, 126.25] | 0.63 | 119.00 [115.50, 126.50] | 120.50 [114.25, 129.50] | 0.80 |
| PLT, ×10^9^/L (median [IQR]) | 269.50 [221.25, 360.50] | 277.50 [218.50, 384.25] | 0.68 | 266.50 [217.00, 321.50] | 289.50 [233.00, 370.25] | 0.07 | 300.00 [246.00, 381.00] | 262.50 [220.25, 343.25] | 0.20 |
| Atelectasis (%) | 80 (31.50) | 72 (28.57) | 0.53 | 23 (32.39) | 23 (33.33) | 1 | 15 (21.43) | 27 (39.13) | 0.04* |
| Pleural effusion (%) | 58 (22.83) | 82 (32.54) | 0.02* | 13 (18.31) | 25 (36.23) | 0.03* | 15 (21.43) | 20 (28.99) | 0.41 |
| Setting (%) |  |  | NA |  |  | NA |  |  | NA |
| Inpatient | 254 (100.00) | 252 (100.00) |  | 71 (100.00) | 69 (100.00) |  | 70 (100.00) | 69 (100.00) |  |
| Outpatient | 0 (0.00) | 0 (0.00) |  | 0 (0.00) | 0 (0.00) |  | 0 (0.00) | 0 (0.00) |  |

Note: Unless otherwise indicated, data are presented as the number of patients, with percentages in parentheses.

Abbreviations: RMPP, refractory *mycoplasma pneumoniae* pneumonia; IQR, Interquartile range; CRP, C-reactive protein; WBC, white blood cell count; NR, neutrophil ratio; MR, monocyte ratio; LR, lymphocyte ratio; BR, basophil ratio; ER, eosinophil ratio; HGB, hemoglobin; PLT, platelet count. *Represents *p* < 0.05.

# 2.5 **Table S5. Characteristics of Patients with Refractory and Non-Refractory *Mycoplasma Pneumonia* Pneumonia in the External Testing Cohort.**

| Variables | External Testing Cohort 1 (n = 331) | | | | External Testing Cohort 2 (n = 108) | | |
| --- | --- | --- | --- | --- | --- | --- | --- |
|  | Non-RMPP (n = 236) | RMPP (n = 95) | | *p*-value | Non-RMPP (n = 55) | RMPP (n = 53) | *p*-value |
| Sex (%) |  |  | | 1 |  |  | 1 |
| Female | 118 (50.00) | 48 (50.53) |  | | 26 (47.27) | 25 (47.17) | 1 |
| Male | 118 (50.00) | 47 (49.47) |  | | 29 (52.73) | 28 (52.83) |  |
| Age, years (median [IQR]) | 7.00 [5.00, 9.00] | 7.00 [5.00, 9.00] | | 0.56 | 11.00 [8.00, 13.50] | 9.00 [7.00, 12.00] | 0.19 |
| Comorbidity (%) | 5 (2.12) | 0 (0.00) | | 0.35 | 5 (9.09) | 2 (3.77) | 0.46 |
| PFD, days (median [IQR]) | 5.00 [4.00, 7.00] | 6.00 [4.00, 7.00] | | 0.16 | 4.00 [1.00, 6.00] | 5.00 [3.00, 8.00] | 0.007* |
| PCD, days (median [IQR]) | 6.00 [4.00, 7.00] | 6.00 [4.00, 8.00] | | 0.78 | 5.00 [3.50, 7.00] | 6.00 [4.00, 8.00] | 0.24 |
| Severe/Critical Pneumonia (%) | 138 (58.47) | 75 (78.95) | | <0.001* | 29 (52.73) | 43 (81.13) | 0.003* |
| ROS (%) | 4 (1.69) | 5 (5.26) | | 0.15 | 4 (7.27) | 6 (11.32) | 0.69 |
| T, ℃ (median [IQR]) | 36.80 [36.50, 37.52] | 37.60 [36.60, 38.45] | | <0.001* | 37.30 [36.92, 37.90] | 37.65 [36.65, 38.58] | 0.16 |
| P, beats/min (mean (SD)) | 102.00 [96.00, 113.00] | 107.00 [96.00, 118.00] | | 0.22 | 106.50 [94.75, 116.00] | 109.00 [98.50, 116.50] | 0.55 |
| R, breaths/min (median [IQR]) | 24.00 [22.00, 26.00] | 24.00 [23.00, 26.00] | | 0.04* | 18.00 [16.25, 20.75] | 18.00 [16.00, 24.00] | 0.59 |
| CRP (mg/L, median [IQR]) | 7.56 [3.05, 14.58] | 7.72 [3.57, 17.85] | | 0.47 | 14.50 [4.00, 32.90] | 20.00 [8.50, 46.00] | 0.06 |
| WBC, ×10^9^/L (median [IQR]) | 7.71 [6.13, 10.07] | 8.24 [6.86, 10.82] | | 0.09 | 7.36 [5.68, 8.85] | 7.34 [5.91, 8.88] | 0.83 |
| NR, % (median [IQR]) | 60.39 [50.50, 68.31] | 58.97 [50.51, 69.25] | | 0.80 | 65.37 [58.61, 72.04] | 67.86 [61.80, 73.19] | 0.42 |
| MR, % (median [IQR]) | 7.21 [5.81, 9.14] | 7.93 [6.32, 9.70] | | 0.22 | 6.69 [5.60, 8.89] | 6.67 [5.15, 7.47] | 0.25 |
| LR, % (median [IQR]) | 30.65 [22.35, 38.23] | 29.53 [21.80, 37.73] | | 0.66 | 23.90 [18.52, 31.98] | 23.03 [17.32, 29.19] | 0.63 |
| BR, % (median [IQR]) | 0.20 [0.13, 0.34] | 0.21 [0.10, 0.34] | | 0.62 | 0.23 [0.16, 0.34] | 0.24 [0.13, 0.35] | 0.90 |
| ER, % (median [IQR]) | 0.97 [0.33, 2.44] | 0.90 [0.22, 2.22] | | 0.48 | 1.24 [0.41, 2.69] | 0.75 [0.20, 2.06] | 0.35 |
| HGB, g/L (median [IQR]) | 125.00 [119.75, 130.00] | 124.00 [119.00, 133.00] | | 0.71 | 132.00 [125.00, 142.00] | 131.00 [121.00, 141.00] | 0.43 |
| PLT, ×10^9^/L (median [IQR]) | 316.00 [260.50, 394.50] | 327.00 [230.00, 396.00] | | 0.56 | 238.00 [196.00, 299.50] | 231.00 [200.00, 309.00] | 0.92 |
| Atelectasis (%) | 2 (0.85) | 5 (5.26) | | 0.04* | 3 (5.45) | 4 (7.55) | 0.96 |
| Pleural effusion (%) | 12 (5.08) | 5 (5.26) | | 0.04* | 2 (3.64) | 16 (30.19) | 0.001* |
| Setting (%) |  |  | | NA |  |  | 0.11 |
| Inpatient | 236 (100.00) | 95 (100.00) | |  | 13 (23.64) | 21 (39.62) |  |
| Outpatient | 0 (0.00) | 0 (0.00) | |  | 42 (76.36) | 32 (60.38) |  |

Note: Unless otherwise indicated, data are presented as the number of patients, with percentages in parentheses.

Abbreviations: RMPP, refractory *mycoplasma pneumoniae* pneumonia; IQR, Interquartile range; PFD, pre-visit fever duration; PCD, pre-visit cough duration; ROS, requirement for oxygen support; T, temperature; P, pulse rate; R, respiratory rate; CRP, C-reactive protein; WBC, white blood cell count; NR, neutrophil ratio; MR, monocyte ratio; LR, lymphocyte ratio; BR, basophil ratio; ER, eosinophil ratio; HGB, hemoglobin; PLT, platelet count. *Represents *p* < 0.05.

# 2.6 **Table S6. Pooled Univariable and Multivariable Logistic Regression Analyses of Clinical Predictors for RMPP in the Training Cohort Across 20 Multiply Imputed Datasets.**

| **Variables** | **Univariate analysis** | | **Multivariate analysis** | |
| --- | --- | --- | --- | --- |
|  | **OR (95% CI)** | ***p*-value** | **OR (95% CI)** | ***p*-value** |
| Sex | 1.10 (0.77–1.56) | 0.60 |  |  |
| Age | 0.94 (0.87–1.00) | 0.06 |  |  |
| Comorbidity | 1.39 (0.79–2.45) | 0.25 |  |  |
| PFD | 1.18 (1.10–1.27) | <0.001* | 1.13 (1.03–1.24) | 0.01* |
| PCD | 1.13 (1.07–1.20) | <0.001* | 1.10 (1.02–1.18) | 0.02* |
| Severe/Critical Pneumonia | 2.47 (1.50–4.05) | <0.001* | 1.88 (1.09–3.23) | 0.02* |
| ROS | 3.85 (2.09–7.07) | <0.001* | 3.25 (1.66–6.35) | <0.001* |
| T | 1.34 (1.10–1.63) | 0.004* | 1.44 (1.12–1.84) | 0.004* |
| P | 1.02 (1.01–1.03) | 0.002* | 1.00 (0.98–1.01) | 0.91 |
| R | 1.06 (1.02–1.09) | <0.001* | 1.01 (0.98–1.05) | 0.47 |
| CRP | 1.01 (1.00–1.02) | 0.045* | 1.01 (1.00–1.02) | 0.09 |
| WBC | 1.07 (1.01–1.13) | 0.02* | 1.01 (0.95–1.08) | 0.67 |
| NR | 1.01 (0.99–1.02) | 0.22 |  |  |
| MR | 1.00 (0.93–1.06) | 0.88 |  |  |
| LR | 0.99 (0.97–1.00) | 0.15 |  |  |
| BR | 1.71 (0.59–4.91) | 0.32 |  |  |
| ER | 1.01 (0.93–1.10) | 0.80 |  |  |
| HGB | 0.98 (0.96–0.99) | 0.01* | 0.98 (0.96–1.00) | 0.06 |
| PLT | 1.00 (1.00–1.00) | 0.47 |  |  |
| Atelectasis | 0.87 (0.59–1.27) | 0.47 |  |  |
| Pleural effusion | 1.63 (1.10–2.42) | 0.02* | 1.30 (0.84–2.02) | 0.24 |

Note: Univariable and multivariable logistic regression analyses were performed separately in each of the 20 imputed datasets, and the resulting estimates were pooled across imputations using Rubin rules. Predictors with a pooled *p* value of less than 0.05 in the univariable analysis were entered into the multivariable model. Abbreviations: OR, Odds ratio; CI, confidence interval; RMPP, refractory mycoplasma pneumoniae pneumonia; PFD, pre-visit fever duration; PCD, pre-visit cough duration; ROS, requirement for oxygen support; T, temperature; P, pulse rate; R, respiratory rate; CRP, C-reactive protein; WBC, white blood cell count; NR, neutrophil ratio; MR, monocyte ratio; LR, lymphocyte ratio; BR, basophil ratio; ER, eosinophil ratio; HGB, hemoglobin; PLT, platelet count. *Represents *p* < 0.05.

# 2.7 **Table S7. Predictive Performance of the Multimodal Nomogram.**

| Dataset | AUC (95% CI) | ACC (95% CI) | SEN (95% CI) | SPE (95% CI) | PPV (95% CI) | NPV (95% CI) | F1 Score (95% CI) |
| --- | --- | --- | --- | --- | --- | --- | --- |
| Training | 0.97 (0.96–0.98) | 0.92 (0.90–0.94) | 0.96 (0.94–0.98) | 0.88 (0.84–0.92) | 0.89 (0.85–0.93) | 0.96 (0.94–0.98) | 0.93 (0.90–0.95) |
| Validation | 0.93 (0.88–0.97) | 0.86 (0.81–0.91) | 0.88 (0.81–0.96) | 0.85 (0.76–0.92) | 0.85 (0.76–0.93) | 0.88 (0.81–0.95) | 0.87 (0.80–0.92) |
| ITC | 0.91 (0.86–0.96) | 0.83 (0.77–0.89) | 0.84 (0.75–0.92) | 0.83 (0.74–0.91) | 0.83 (0.74–0.91) | 0.84 (0.75–0.92) | 0.83 (0.76–0.89) |
| ETC1 | 0.89 (0.85–0.94) | 0.85 (0.81–0.89) | 0.83 (0.75–0.90) | 0.86 (0.81–0.90) | 0.70 (0.61–0.78) | 0.93 (0.89–0.96) | 0.76 (0.69–0.82) |
| ETC2 | 0.89 (0.83–0.96) | 0.85 (0.78–0.92) | 0.77 (0.65–0.88) | 0.93 (0.85–0.98) | 0.91 (0.82–0.98) | 0.81 (0.71–0.90) | 0.84 (0.75–0.91) |

Note: Abbreviations: AUC, area under the curve; ACC, accuracy; SEN, sensitivity; SPE, specificity; PPV, positive prediction value; NPV, negative prediction value; CI, confidence interval; ITC, internal testing cohort; ETC1, external testing cohort 1; ETC2, external testing cohort 2.

# 2.8 **Table S8. Absolute Classification Counts for the Predictive Models Across the Study Cohorts.**

| **Dataset** | **Model** | **TP** | **TN** | **FP** | **FN** |
| --- | --- | --- | --- | --- | --- |
| **Training** | Clinical | 147 | 191 | 63 | 105 |
|  | Trans-DLF | 250 | 150 | 104 | 2 |
|  | 3D-CNN | 242 | 222 | 32 | 10 |
|  | Multimodal Nomogram | 243 | 224 | 30 | 9 |
| **Validation** | Clinical | 45 | 55 | 16 | 24 |
|  | Trans-DLF | 58 | 54 | 17 | 11 |
|  | 3D-CNN | 60 | 59 | 12 | 9 |
|  | Multimodal Nomogram | 61 | 60 | 11 | 8 |
| **ITC** | Clinical | 47 | 55 | 15 | 22 |
|  | Trans-DLF | 61 | 35 | 35 | 8 |
|  | 3D-CNN | 58 | 57 | 13 | 11 |
|  | Multimodal Nomogram | 58 | 58 | 12 | 11 |
| **ETC1** | Clinical | 26 | 208 | 28 | 69 |
|  | Trans-DLF | 74 | 192 | 44 | 21 |
|  | 3D-CNN | 80 | 192 | 44 | 15 |
|  | Multimodal Nomogram | 79 | 202 | 34 | 16 |
| **ETC2** | Clinical | 16 | 47 | 8 | 37 |
|  | Trans-DLF | 40 | 43 | 12 | 13 |
|  | 3D-CNN | 44 | 46 | 9 | 9 |
|  | Multimodal Nomogram | 41 | 51 | 4 | 12 |

Note: Absolute classification counts were calculated using the model-specific cutoff values determined by maximizing the Youden index in the validation cohort. RMPP was defined as the positive class. Abbreviations: TP, true positive; TN, true negative; FP, false positive; FN, false negative; RMPP, refractory Mycoplasma pneumoniae pneumonia; trans-DLF, transformer-based deep learning framework; 3D-CNN, three-dimensional convolutional neural network.

# 2.9 **Table S9. Pooled Performance of the Clinical and Multimodal Models Across 20 Multiply Imputed Datasets.**

| **Models** | **AUC (95% CI)** | **ACC (95% CI)** | **SEN (95% CI)** | **SPE (95% CI)** | **PPV (95% CI)** | **NPV (95% CI)** | **F1 Score (95% CI)** |
| --- | --- | --- | --- | --- | --- | --- | --- |
| Clinical | |  |  |  |  |  |  |
| Training | 0.71 (0.67–0.76) | 0.67 (0.63–0.71) | 0.65 (0.59–0.71) | 0.69 (0.63–0.74) | 0.67 (0.63–0.72) | 0.66 (0.62–0.71) | 0.66 (0.61–0.70) |
| Validation | 0.73 (0.64–0.81) | 0.70 (0.62–0.77) | 0.67 (0.54–0.77) | 0.73 (0.61–0.83) | 0.71 (0.61–0.79) | 0.69 (0.61–0.77) | 0.69 (0.59–0.77) |
| ITC | 0.77 (0.68–0.84) | 0.72 (0.64–0.79) | 0.72 (0.60–0.82) | 0.72 (0.60–0.82) | 0.72 (0.63–0.80) | 0.73 (0.64–0.80) | 0.72 (0.64–0.79) |
| ETC1 | 0.69 (0.62–0.75) | 0.71 (0.66–0.75) | 0.35 (0.26–0.45) | 0.85 (0.80–0.89) | 0.49 (0.38–0.59) | 0.76 (0.73–0.79) | 0.41 (0.32–0.50) |
| ETC2 | 0.67 (0.56–0.76) | 0.60 (0.51–0.68) | 0.41 (0.28–0.55) | 0.78 (0.64–0.88) | 0.64 (0.49–0.78) | 0.58 (0.51–0.64) | 0.50 (0.37–0.63) |
| Multimodal Nomogram | |  |  |  |  |  |  |
| Training | 0.97 (0.96–0.98) | 0.90 (0.87–0.93) | 0.96 (0.93–0.98) | 0.84 (0.79–0.88) | 0.86 (0.82–0.89) | 0.96 (0.92–0.98) | 0.91 (0.88–0.93) |
| Validation | 0.93 (0.87–0.96) | 0.86 (0.80–0.91) | 0.93 (0.64–0.99) | 0.80 (0.69–0.88) | 0.82 (0.73–0.88) | 0.92 (0.62–0.99) | 0.87 (0.81–0.91) |
| ITC | 0.91 (0.84–0.95) | 0.86 (0.79–0.91) | 0.90 (0.76–0.96) | 0.83 (0.71–0.90) | 0.84 (0.75–0.90) | 0.89 (0.76–0.96) | 0.87 (0.80–0.91) |
| ETC1 | 0.90 (0.85–0.93) | 0.82 (0.77–0.86) | 0.85 (0.76–0.91) | 0.80 (0.75–0.85) | 0.63 (0.57–0.70) | 0.93 (0.89–0.96) | 0.73 (0.67–0.78) |
| ETC2 | 0.90 (0.81–0.95) | 0.81 (0.72–0.87) | 0.83 (0.69–0.92) | 0.79 (0.65–0.88) | 0.79 (0.68–0.87) | 0.83 (0.71–0.91) | 0.81 (0.72–0.88) |

Note: The clinical and multimodal models were fitted and evaluated separately in each of the 20 imputed datasets. Performance estimates were pooled across imputations using Rubin rules. Metrics bounded between 0 and 1 were pooled on the logit scale and subsequently back-transformed. Stratified bootstrap resampling with 2000 replicates was used to estimate within-imputation variance. Abbreviations: Trans-DLF, Transformer-based deep learning framework; 3D-CNN, 3D convolutional neural network; AUC, area under the curve; ACC, accuracy; SEN, sensitivity; SPE, specificity; PPV, positive prediction value; NPV, negative prediction value; CI, confidence interval; ITC, internal testing cohort; ETC1, external testing cohort 1; ETC2, external testing cohort 2.

# 2.10 **Table S10. Pooled DeLong Test Results for AUC Comparisons Across 20 Multiply Imputed Datasets.**

| **Dataset** | **Comparison** | **AUC difference (95% CI)** | **Bonferroni-adjusted *P* value** |
| --- | --- | --- | --- |
| **Training** | Trans-DLF vs Clinical | 0.25 (0.21–0.30) | <0.001* |
| **Training** | Trans-DLF vs 3D-CNN | 0.02 (0.00–0.03) | 0.052 |
| **Training** | Trans-DLF vs Nomogram | 0.00 (-0.01–0.01) | 1.00 |
| **Validation** | Trans-DLF vs Clinical | 0.18 (0.08–0.27) | <0.001* |
| **Validation** | Trans-DLF vs 3D-CNN | 0.04 (-0.04–0.11) | 0.92 |
| **Validation** | Trans-DLF vs Nomogram | -0.02 (-0.03–-0.00) | 0.11 |
| **ITC** | Trans-DLF vs Clinical | 0.13 (0.04–0.22) | 0.02* |
| **ITC** | Trans-DLF vs 3D-CNN | 0.03 (-0.04–0.10) | 1.00 |
| **ITC** | Trans-DLF vs Nomogram | -0.01 (-0.03–0.02) | 1.00 |
| **ETC1** | Trans-DLF vs Clinical | 0.20 (0.12–0.28) | <0.001* |
| **ETC1** | Trans-DLF vs 3D-CNN | 0.07 (-0.00–0.13) | 0.18 |
| **ETC1** | Trans-DLF vs Nomogram | -0.01 (-0.03–0.01) | 1.00 |
| **ETC2** | Trans-DLF vs Clinical | 0.22 (0.10–0.34) | 0.001* |
| **ETC2** | Trans-DLF vs 3D-CNN | 0.05 (-0.04–0.15) | 0.84 |
| **ETC2** | Trans-DLF vs Nomogram | -0.01 (-0.04–0.01) | 1.00 |

**NOTE:** Within each cohort, pairwise differences in AUC and the corresponding DeLong variances were estimated separately in each of the 20 imputed datasets. The resulting estimates were pooled across imputations using Rubin rules to account for both within-imputation and between-imputation variability. Abbreviations: Trans-DLF, Transformer-based deep learning framework; 3D-CNN, 3D convolutional neural network; AUC, area under the curve; CI, confidence interval; ITC, internal testing cohort; ETC1, external testing cohort 1; ETC2, external testing cohort 2. *Represents *p* < 0.05.

# 2.11 **Table S11. The prediction performance of Trans-DLF in the Subgroup Analysis.**

| Dataset | Subgroups | RMPP | AUC (95% CI) | ACC (95% CI) | SEN (95% CI) | SPE (95% CI) | PPV (95% CI) | NPV (95% CI) | F1 Score (95% CI) |
| --- | --- | --- | --- | --- | --- | --- | --- | --- | --- |
| ETC | Age<5 years | 24/57 | 0.87 (0.77-0.98) | 0.81 (0.70-0.90) | 0.83 (0.67-0.96) | 0.79 (0.64-0.92) | 0.74 (0.56-0.90) | 0.87 (0.73-0.97) | 0.78 (0.64-0.89) |
|  | 5≤Age≤12 years | 106/333 | 0.90 (0.86-0.94) | 0.84 (0.80-0.87) | 0.85 (0.78-0.91) | 0.83 (0.78-0.88) | 0.70 (0.62-0.78) | 0.92 (0.88-0.96) | 0.77 (0.70-0.83) |
|  | Age>12 years | 18/49 | 0.81 (0.66-0.96) | 0.76 (0.63-0.88) | 0.78 (0.58-0.95) | 0.74 (0.59-0.89) | 0.64 (0.41-0.84) | 0.85 (0.70-0.97) | 0.70 (0.51-0.85) |
|  | CRP<cutoff | 96/307 | 0.89 (0.84-0.94) | 0.83 (0.79-0.87) | 0.86 (0.79-0.93) | 0.82 (0.77-0.87) | 0.69 (0.60-0.77) | 0.93 (0.89-0.96) | 0.77 (0.70-0.82) |
|  | CRP≥cutoff | 48/117 | 0.85 (0.78-0.93) | 0.79 (0.71-0.85) | 0.77 (0.64-0.88) | 0.80 (0.70-0.89) | 0.73 (0.60-0.84) | 0.83 (0.74-0.91) | 0.75 (0.64-0.83) |
|  | PFD<cutoff | 90/306 | 0.87 (0.82-0.92) | 0.82 (0.77-0.86) | 0.80 (0.72-0.88) | 0.82 (0.77-0.88) | 0.65 (0.57-0.74) | 0.91 (0.86-0.95) | 0.72 (0.65-0.79) |
|  | PFD≥cutoff | 58/133 | 0.92 (0.86-0.97) | 0.84 (0.77-0.90) | 0.90 (0.81-0.97) | 0.80 (0.71-0.89) | 0.78 (0.68-0.87) | 0.91 (0.83-0.97) | 0.83 (0.76-0.90) |
|  | NR<cutoff | 75/234 | 0.89 (0.84-0.94) | 0.82 (0.77-0.86) | 0.84 (0.75-0.92) | 0.81 (0.74-0.87) | 0.67 (0.57-0.76) | 0.91 (0.86-0.96) | 0.75 (0.67-0.81) |
|  | NR≥cutoff | 51/123 | 0.92 (0.87-0.98) | 0.89 (0.84-0.94) | 0.88 (0.78-0.96) | 0.90 (0.83-0.97) | 0.86 (0.77-0.96) | 0.92 (0.85-0.97) | 0.87 (0.80-0.94) |
|  | Outpatient | 32/74 | 0.87 (0.78-0.96) | 0.81 (0.72-0.89) | 0.78 (0.63-0.91) | 0.83 (0.70-0.94) | 0.78 (0.63-0.91) | 0.83 (0.71-0.94) | 0.78 (0.65-0.88) |
| ITC | Age<5 years | 19/45 | 0.92 (0.84-1.00) | 0.82 (0.71-0.93) | 0.84 (0.67-1.00) | 0.81 (0.65-0.96) | 0.76 (0.58-0.94) | 0.88 (0.72-1.00) | 0.80 (0.65-0.92) |
|  | 5≤Age≤12 years | 50/94 | 0.89 (0.83-0.96) | 0.83 (0.76-0.90) | 0.84 (0.73-0.93) | 0.82 (0.70-0.93) | 0.84 (0.73-0.93) | 0.82 (0.69-0.92) | 0.84 (0.75-0.91) |
|  | CRP<cutoff | 46/92 | 0.89 (0.82-0.95) | 0.80 (0.72-0.89) | 0.85 (0.74-0.94) | 0.76 (0.63-0.89) | 0.78 (0.66-0.89) | 0.83 (0.71-0.94) | 0.81 (0.71-0.89) |
|  | CRP≥cutoff | 20/41 | 0.89 (0.79-0.99) | 0.88 (0.78-0.98) | 0.80 (0.61-0.95) | 0.95 (0.84-1.00) | 0.94 (0.81-1.00) | 0.83 (0.67-0.96) | 0.86 (0.73-0.97) |
|  | PFD<cutoff | 31/80 | 0.91 (0.84-0.98) | 0.81 (0.72-0.90) | 0.81 (0.66-0.93) | 0.82 (0.71-0.92) | 0.73 (0.59-0.88) | 0.87 (0.77-0.96) | 0.77 (0.65-0.88) |
|  | PFD≥cutoff | 38/59 | 0.88 (0.79-0.97) | 0.85 (0.75-0.93) | 0.87 (0.75-0.97) | 0.81 (0.62-0.96) | 0.89 (0.78-0.97) | 0.77 (0.58-0.95) | 0.88 (0.79-0.95) |
|  | NR<cutoff | 37/76 | 0.93 (0.87-0.98) | 0.84 (0.75-0.92) | 0.89 (0.78-0.97) | 0.80 (0.66-0.91) | 0.81 (0.68-0.92) | 0.89 (0.77-0.97) | 0.85 (0.75-0.92) |
|  | NR≥cutoff | 29/57 | 0.86 (0.77-0.96) | 0.81 (0.70-0.91) | 0.76 (0.60-0.90) | 0.86 (0.72-0.97) | 0.85 (0.70-0.97) | 0.77 (0.61-0.91) | 0.80 (0.67-0.90) |
| Validation | Age<5 years | 21/50 | 0.97 (0.94-1.00) | 0.90 (0.80-0.98) | 0.95 (0.85-1.00) | 0.86 (0.71-0.97) | 0.83 (0.67-0.96) | 0.96 (0.87-1.00) | 0.89 (0.77-0.98) |
|  | 5≤Age≤12 years | 48/90 | 0.88 (0.81-0.95) | 0.82 (0.74-0.90) | 0.83 (0.72-0.93) | 0.81 (0.68-0.92) | 0.83 (0.73-0.94) | 0.81 (0.68-0.92) | 0.83 (0.75-0.91) |
|  | CRP<cutoff | 47/98 | 0.92 (0.86-0.97) | 0.85 (0.78-0.91) | 0.87 (0.77-0.96) | 0.82 (0.71-0.93) | 0.82 (0.71-0.92) | 0.88 (0.78-0.96) | 0.84 (0.76-0.91) |
|  | CRP≥cutoff | 21/38 | 0.90 (0.79-1.00) | 0.84 (0.71-0.95) | 0.86 (0.68-1.00) | 0.82 (0.62-1.00) | 0.86 (0.70-1.00) | 0.82 (0.60-1.00) | 0.86 (0.72-0.95) |
|  | PFD<cutoff | 30/81 | 0.89 (0.80-0.97) | 0.84 (0.75-0.91) | 0.87 (0.73-0.97) | 0.82 (0.72-0.92) | 0.74 (0.60-0.88) | 0.91 (0.82-0.98) | 0.80 (0.69-0.89) |
|  | PFD≥cutoff | 39/59 | 0.94 (0.89-1.00) | 0.86 (0.78-0.95) | 0.87 (0.76-0.97) | 0.85 (0.67-1.00) | 0.92 (0.82-1.00) | 0.77 (0.59-0.94) | 0.90 (0.81-0.96) |
|  | NR<cutoff | 33/79 | 0.92 (0.86-0.98) | 0.83 (0.75-0.91) | 0.91 (0.80-1.00) | 0.78 (0.66-0.90) | 0.75 (0.61-0.88) | 0.92 (0.83-1.00) | 0.82 (0.71-0.91) |
|  | NR≥cutoff | 35/57 | 0.91 (0.84-0.99) | 0.86 (0.77-0.95) | 0.83 (0.70-0.94) | 0.91 (0.77-1.00) | 0.94 (0.84-1.00) | 0.77 (0.59-0.92) | 0.88 (0.79-0.95) |
| Training | Age<5 years | 85/149 | 0.96 (0.93-0.99) | 0.91 (0.87-0.95) | 0.98 (0.94-1.00) | 0.83 (0.73-0.92) | 0.88 (0.81-0.95) | 0.96 (0.91-1.00) | 0.93 (0.88-0.96) |
|  | 5≤Age≤12 years | 163/349 | 0.97 (0.96-0.99) | 0.92 (0.89-0.95) | 0.95 (0.92-0.98) | 0.89 (0.84-0.93) | 0.88 (0.83-0.93) | 0.95 (0.92-0.98) | 0.91 (0.88-0.94) |
|  | CRP<cutoff | 159/340 | 0.97 (0.96-0.99) | 0.92 (0.88-0.94) | 0.97 (0.94-0.99) | 0.87 (0.82-0.91) | 0.86 (0.81-0.91) | 0.97 (0.94-0.99) | 0.91 (0.88-0.94) |
|  | CRP≥cutoff | 85/142 | 0.97 (0.95-0.99) | 0.92 (0.87-0.96) | 0.94 (0.89-0.99) | 0.88 (0.78-0.95) | 0.92 (0.86-0.97) | 0.91 (0.83-0.98) | 0.93 (0.89-0.97) |
|  | PFD<cutoff | 115/283 | 0.96 (0.94-0.98) | 0.91 (0.87-0.94) | 0.94 (0.89-0.98) | 0.89 (0.84-0.93) | 0.85 (0.79-0.91) | 0.95 (0.92-0.99) | 0.89 (0.85-0.93) |
|  | PFD≥cutoff | 137/223 | 0.98 (0.96-0.99) | 0.93 (0.89-0.96) | 0.98 (0.95-1.00) | 0.85 (0.77-0.92) | 0.91 (0.86-0.95) | 0.96 (0.91-1.00) | 0.94 (0.91-0.97) |
|  | NR<cutoff | 135/285 | 0.96 (0.94-0.98) | 0.90 (0.86-0.93) | 0.94 (0.90-0.98) | 0.86 (0.80-0.92) | 0.86 (0.80-0.91) | 0.94 (0.90-0.98) | 0.90 (0.86-0.93) |
|  | NR≥cutoff | 109/197 | 0.98 (0.97-1.00) | 0.94 (0.90-0.97) | 0.98 (0.95-1.00) | 0.89 (0.82-0.95) | 0.91 (0.86-0.96) | 0.97 (0.94-1.00) | 0.95 (0.92-0.97) |

Note: the cutoff values for PFD, CRP, and NR were 6.5, 18.44, and 67.34% respectively. Abbreviations: RMPP, Refractory *Mycoplasma Pneumonia* Pneumonia; AUC, area under the curve; ACC, accuracy; SEN, sensitivity; SPE, specificity; PPV, positive prediction value; NPV, negative prediction value; CI, confidence interval; ITC, internal testing cohort; ETC1, external testing cohort 1; ETC2, external testing cohort 2; Trans-DLF, Transformer-based deep learning framework; PFD, pre-visit fever duration; CRP, C-reactive Protein; NR, neutrophil ratio.

# 2.12 **Table S12. Performance of the trans-DLF at clinically oriented operating thresholds.**

| Cohort | Operating threshold | Cutoff |  | | Performance, estimate (95% CI) | | | | | |
| --- | --- | --- | --- | --- | --- | --- | --- | --- | --- | --- |
|  |  |  | **FP** | **FN** | **Accuracy** | **Sensitivity** | **Specificity** | **PPV** | **NPV** | **F1 Score** |
| Training cohort | High-sensitivity | 0.45 | 59 | 8 | 0.87 (0.84–0.90) | 0.97 (0.94–0.99) | 0.77 (0.71–0.82) | 0.81 (0.77–0.84) | 0.96 (0.93–0.98) | 0.88 (0.86–0.90) |
|  | High-specificity | 0.72 | 27 | 17 | 0.91 (0.89–0.93) | 0.93 (0.90–0.96) | 0.89 (0.85–0.93) | 0.90 (0.86–0.93) | 0.93 (0.90–0.96) | 0.91 (0.89–0.94) |
| Validation cohort | High-sensitivity | 0.45 | 19 | 6 | 0.82 (0.76–0.88) | 0.91 (0.84–0.97) | 0.73 (0.63–0.83) | 0.77 (0.70–0.84) | 0.90 (0.82–0.96) | 0.83 (0.78–0.88) |
|  | High-specificity | 0.72 | 7 | 15 | 0.84 (0.79–0.90) | 0.78 (0.68–0.88) | 0.90 (0.83–0.96) | 0.89 (0.81–0.96) | 0.81 (0.74–0.88) | 0.83 (0.76–0.90) |
| Internal testing cohort | High-sensitivity | 0.45 | 16 | 8 | 0.83 (0.76–0.88) | 0.88 (0.80–0.96) | 0.77 (0.67–0.87) | 0.79 (0.72–0.87) | 0.87 (0.80–0.95) | 0.84 (0.78–0.89) |
|  | High-specificity | 0.72 | 8 | 15 | 0.83 (0.77–0.89) | 0.78 (0.68–0.87) | 0.89 (0.81–0.96) | 0.87 (0.80–0.95) | 0.81 (0.73–0.88) | 0.82 (0.75–0.89) |
| External testing cohort 1 | High-sensitivity | 0.45 | 61 | 12 | 0.78 (0.73–0.82) | 0.87 (0.80–0.94) | 0.74 (0.69–0.80) | 0.58 (0.52–0.63) | 0.94 (0.90–0.97) | 0.69 (0.64–0.74) |
|  | High-specificity | 0.72 | 26 | 22 | 0.85 (0.82–0.89) | 0.77 (0.68–0.85) | 0.89 (0.85–0.93) | 0.74 (0.67–0.82) | 0.91 (0.87–0.94) | 0.75 (0.69–0.81) |
| External testing cohort 2 | High-sensitivity | 0.45 | 11 | 9 | 0.81 (0.74–0.89) | 0.83 (0.72–0.92) | 0.80 (0.69–0.89) | 0.80 (0.72–0.89) | 0.83 (0.75–0.92) | 0.81 (0.74–0.89) |
|  | High-specificity | 0.72 | 6 | 12 | 0.83 (0.76–0.90) | 0.77 (0.66–0.89) | 0.89 (0.80–0.96) | 0.87 (0.78–0.96) | 0.80 (0.72–0.88) | 0.82 (0.73–0.89) |
| Outpatient subset | High-sensitivity | 0.45 | 8 | 7 | 0.80 (0.70–0.89) | 0.78 (0.62–0.91) | 0.81 (0.69–0.93) | 0.76 (0.65–0.89) | 0.83 (0.73–0.93) | 0.77 (0.66–0.87) |
|  | High-specificity | 0.72 | 4 | 9 | 0.82 (0.74–0.91) | 0.72 (0.56–0.88) | 0.90 (0.81–0.98) | 0.85 (0.73–0.96) | 0.81 (0.72–0.90) | 0.78 (0.65–0.89) |

Note: Values for performance metrics are estimates with 95% CIs in parentheses. The high-sensitivity and high-specificity thresholds were selected in the validation cohort and applied unchanged to the training, internal testing, and external testing cohorts. RMPP was considered the positive class. Abbreviations: AUC, area under the receiver operating characteristic curve; CI, confidence interval; FN, false negative; FP, false positive; NPV, negative predictive value; PPV, positive predictive value; RMPP, refractory *Mycoplasma pneumoniae* pneumonia; TN, true negative; TP, true positive; Trans-DLF, Transformer-based deep learning framework.

# 3 **Supplementary Figure**

# 3.1 **Figure S1. Distribution of Patient Age in the Center 1.**

Note: This density curve represents the age structure of the center 1, illustrating the relative proportion of participants across different age groups.

# 3.2 **Figure S2. Multimodal nomogram for predicting refractory *Mycoplasma pneumoniae* pneumonia (RMPP).**

Note: The nomogram integrates key clinical predictors and the predicted probability from the Transformer-based deep learning framework to estimate the individual risk of RMPP. Abbreviations: PFD, pre-visit fever duration; PCD, pre-visit cough duration; ROS, requirement for oxygen support; T, temperature; HGB, hemoglobin, RMPP, refractory *Mycoplasma pneumoniae* pneumonia; Trans-DLF, Transformer-based deep learning framework.

# 3.3 **Figure S3. Multimodal Nomogram Constructed Using Pooled Regression Coefficients Across 20 Multiply Imputed Datasets.**

Note: The multimodal nomogram was constructed using the regression coefficients pooled across the 20 imputed training datasets according to Rubin rules. The model incorporated the clinical predictors retained in the multiple-imputation analysis together with the probability from the Transformer-based deep learning framework. Abbreviations: PFD, pre-visit fever duration; PCD, pre-visit cough duration; ROS, requirement for oxygen support; T, temperature; RMPP, refractory *Mycoplasma pneumoniae* pneumonia; Trans-DLF, Transformer-based deep learning framework.

# 3.4 **Figure S4. Predictive model performance for refractory *Mycoplasma pneumoniae* pneumonia (RMPP) in Training Cohort.**

Note: (A) Receiver operating characteristic (ROC) curves comparing the Transformer-based deep learning framework (Trans-DLF), the 3D convolutional neural network (3D-CNN), the clinical model, and the multimodal nomogram in the training cohort. (B) Waterfall plots illustrating the predicted probabilities of RMPP generated by the Trans-DLF in the training cohort. An optimal cutoff of 0.52 (the horizontal baseline) was used to classify cases. Blue bars below the baseline represent correctly classified non-RMPP cases. Orange bars above the baseline indicate correctly classified RMPP cases. Bars that cross the baseline represent misclassified cases.

Abbreviations: Trans-DLF, Transformer-based deep learning framework; 3D-CNN, 3D convolutional neural network; AUC, area under the curve.

# 4 **Supplementary Result**

# 4.1 **Results of the Multiple-Imputation Sensitivity Analysis**

In the pooled univariable analyses of the 20 multiply imputed datasets, the overall associations between the candidate clinical predictors and RMPP were consistent with those observed in the primary analysis. In the pooled multivariable analysis, five of the six predictors included in the primary clinical model remained statistically significant. HGB, which showed borderline significance in the primary analysis (*P* = 0.048), was no longer independently associated with RMPP after between-imputation uncertainty was incorporated. The directions and magnitudes of the effect estimates for the remaining predictors were comparable to those obtained in the primary analysis (Supplementary Table S6).

The clinical and multimodal models were subsequently reconstructed in each of the 20 imputed datasets. Across the training, validation, internal testing, and external testing cohorts, the pooled estimates of discrimination and threshold-dependent performance were comparable to those obtained in the primary analysis (Supplementary Table S9).

The pooled DeLong analyses also yielded conclusions consistent with those of the primary analysis. The trans-DLF continued to show significantly greater discrimination than the clinical model (all *p* < 0.001 vs. the trans-DLF), whereas no statistically significant differences in AUC were observed between the trans-DLF and the multimodal model. Thus, accounting for both sampling variability and between-imputation uncertainty did not materially alter the principal conclusions of the model comparisons (Supplementary Table S10).
